# Screening of differential gene expression patterns through survival analysis for diagnosis, prognosis and therapies of clear cell renal cell carcinoma

**DOI:** 10.1371/journal.pone.0310843

**Published:** 2024-09-30

**Authors:** Alvira Ajadee, Sabkat Mahmud, Md. Bayazid Hossain, Reaz Ahmmed, Md. Ahad Ali, Md. Selim Reza, Saroje Kumar Sarker, Md. Nurul Haque Mollah

**Affiliations:** 1 Department of Statistics, Bioinformatics Lab (Dry), University of Rajshahi, Rajshahi, Bangladesh; 2 Department of Agricultural and Applied Statistics, Bangladesh Agricultural University, Mymensingh, Bangladesh; 3 Department of Biochemistry & Molecular Biology, University of Rajshahi, Rajshahi, Bangladesh; 4 Department of Chemistry, University of Rajshahi, Rajshahi, Bangladesh; 5 Center for Biomedical Informatics & Genomics, School of Medicine, Tulane University, New Orleans, LA, United States of America; Memorial Sloan Kettering Cancer Center, UNITED STATES OF AMERICA

## Abstract

Clear cell renal cell carcinoma (ccRCC) is the most prevalent subtype of kidney cancer. Although there is increasing evidence linking ccRCC to genetic alterations, the exact molecular mechanism behind this relationship is not yet completely known to the researchers. Though drug therapies are the best choice after the metastasis, unfortunately, the majority of the patients progressively develop resistance against the therapeutic drugs after receiving it for almost 2 years. In this case, multi-targeted different variants of therapeutic drugs are essential for effective treatment against ccRCC. To understand molecular mechanisms of ccRCC development and progression, and explore multi-targeted different variants of therapeutic drugs, it is essential to identify ccRCC-causing key genes (KGs). In order to obtain ccRCC-causing KGs, at first, we detected 133 common differentially expressed genes (cDEGs) between ccRCC and control samples based on nine (9) microarray gene-expression datasets with NCBI accession IDs GSE16441, GSE53757, GSE66270, GSE66272, GSE16449, GSE76351, GSE66271, GSE71963, and GSE36895. Then, we filtered these cDEGs through survival analysis with the independent TCGA and GTEx database and obtained 54 scDEGs having significant prognostic power. Next, we used protein-protein interaction (PPI) network analysis with the reduced set of 54 scDEGs to identify ccRCC-causing top-ranked eight KGs (*PLG*, *ENO2*, *ALDOB*, *UMOD*, *ALDH6A1*, *SLC12A3*, *SLC12A1*, *SERPINA5*). The pan-cancer analysis with KGs based on TCGA database showed the significant association with different subtypes of kidney cancers including ccRCC. The gene regulatory network (GRN) analysis revealed some crucial transcriptional and post-transcriptional regulators of KGs. The scDEGs-set enrichment analysis significantly identified some crucial ccRCC-causing molecular functions, biological processes, cellular components, and pathways that are linked to the KGs. The results of DNA methylation study indicated the hypomethylation and hyper-methylation of KGs, which may lead the development of ccRCC. The immune infiltrating cell types (CD8+ T and CD4+ T cell, B cell, neutrophil, dendritic cell and macrophage) analysis with KGs indicated their significant association in ccRCC, where KGs are positively correlated with CD4+ T cells, but negatively correlated with the majority of other immune cells, which is supported by the literature review also. Then we detected 10 repurposable drug molecules (Irinotecan, Imatinib, Telaglenastat, Olaparib, RG-4733, Sorafenib, Sitravatinib, Cabozantinib, Abemaciclib, and Dovitinib.) by molecular docking with KGs-mediated receptor proteins. Their ADME/T analysis and cross-validation with the independent receptors, also supported their potent against ccRCC. Therefore, these outputs might be useful inputs/resources to the wet-lab researchers and clinicians for considering an effective treatment strategy against ccRCC.

## 1. Introduction

**Fig 1:**
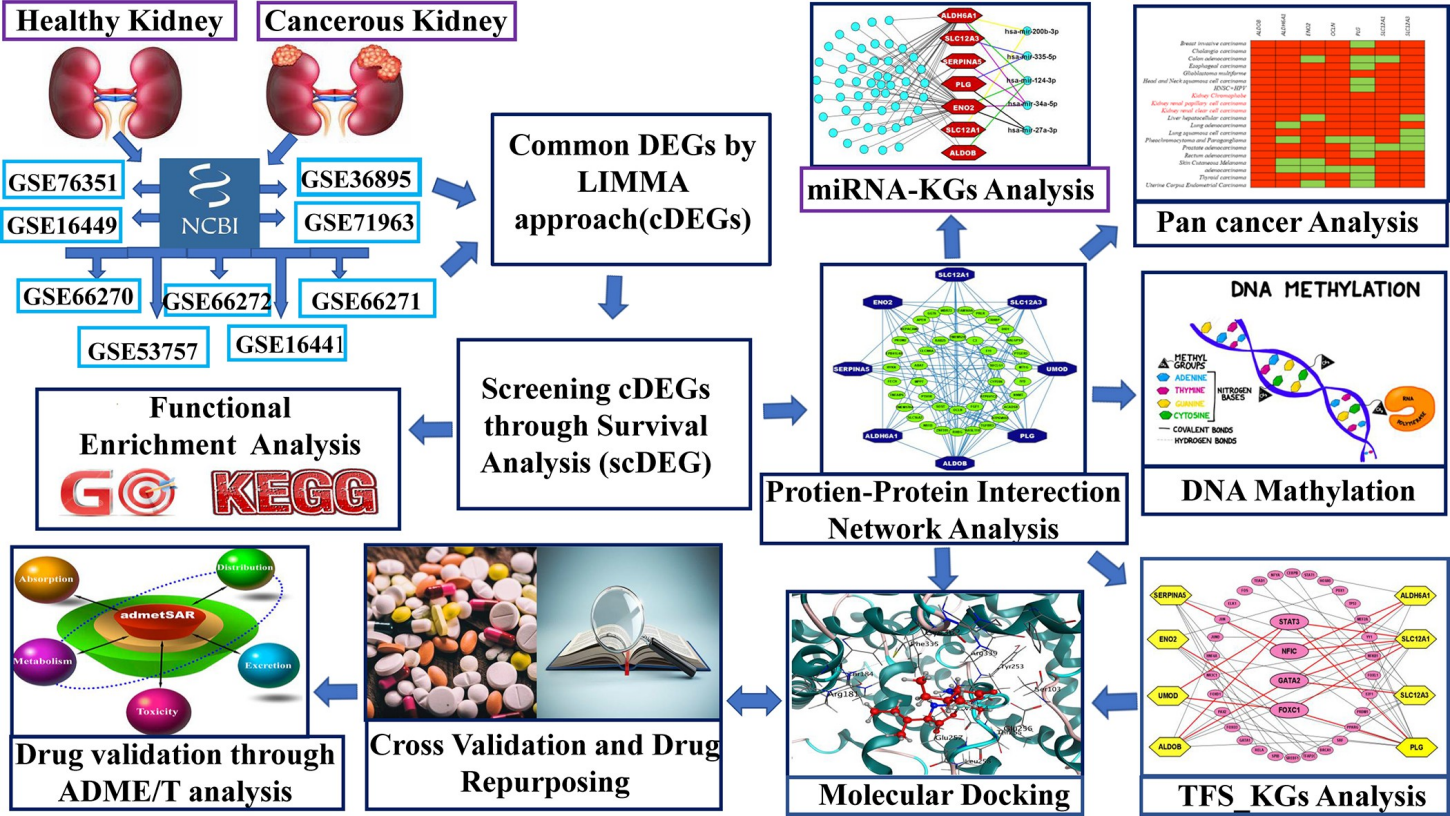
The study’s workflow.

Renal carcinoma, a common urinary tract tumor, accounts for 2% to 3% of adult cancers [1]. Clear cell renal cell carcinomas (ccRCC) account for 70–85% of all cases of renal cell carcinomas (RCC), which make up 90% of cases overall [2]. There are four pathological phases of ccRCC, ranging from grade I to grade IV, where the survival rate of 20% grade IV patients is more than 5-years [3]. With RCC patients responding poorly to traditional radiation and chemotherapy, surgical excision remains the most effective therapeutic option [4]. However, drug therapies would be the best choice after the metastasis [5]. Unfortunately, the majority of the patients progressively develop resistance against the therapeutic drugs after receiving it for almost 2 years [6]. Moreover, the combination of ICIs (immune checkpoint inhibitors) with anti-VEGF (vascular endothelial growth factor) drugs for the treatment of renal cancer may increase the risk of cardiovascular events, according to a meta-analysis. This emphasizes the need for more research in this therapeutic setting [7]. In this case, multi-targeted different variants of therapeutic drugs may be essential for effective treatment against ccRCC to increase the survival length of patients [8,9]. The therapy of RCC can be improved by integrating Artificial Intelligence (AI), sophisticated imaging, and genomics [10,11]. To understand molecular mechanisms of ccRCC development and progression, and explore multi-targeted different variants of therapeutic drugs, it is essential to identify ccRCC-causing key genes (molecular signatures). Nevertheless, it is very difficult to explore top-ranked KGs and candidate therapeutic agents from huge number of alternatives through the wet-lab experiments only, since wet-lab experiments are time consuming, laborious and costly. To overcome this issues, bioinformatics and system biology approaches playing the significant roles [12–17]. There are several studies that explored ccRCC-causing key genes (KGs) [18–36]. However, their KGs-sets based on single transcriptomics dataset were not so consistent [18,19,29]. It may have occurred as a result of environmental and geographical differences in the datasets. To identify KGs-set that is more reliable and efficient, some researchers analyzed multiple transcriptomics datasets [20,21,30–36]. As for example, some individual studies considered two datasets [30,34,35], three datasets [20–24,36] and at most four datasets [25–28] for exploring KGs, respectively. Few of them, nonetheless, investigated the KGs-set guided drug compounds they had suggested for the therapy of ccRCC [25,28,30]. None of them have yet examined the efficacy of their recommended therapeutic compounds against the separate KGs that are proposed by other studies to cause ccRCC. Therefore, in this study, we considered nine transcriptomics datasets to explore ccRCC causing KGs more accurately for diagnosis, prognosis and therapies. To find ccRCC-causing KGs based on nine datasets, first, for each dataset, we must determine which genes are differentially expressed (DEGs) between the ccRCC and control samples. Then we have to find the common DEGs (cDEGs)-set from the nine DEGs-sets. To make the cDEGs-set more reliable and effective, we have to screening cDEGs-set through the survival analysis based on independent TCGA database [37]. Then the reduced cDEGs-set will be used to find the ccRCC-causing key genes (KGs) through their protein-protein interaction (PPI) network [38] analysis. To verify these KGs as the ccRCC-causing genes through other independent database, we have to performed their pan-cancer [39] analysis. To investigate the pathogenetic processes of KGs, we have to performed GO functional [40] and KEGG [41] pathway enrichment analysis, DNA methylation analysis[42] and gene regulatory network analysis (GRN) [43]. In order to explore, KGs-guided candidate drug molecules, we have to consider molecular docking analysis. We must take into consideration molecular docking studies once again in order to confirm the effectiveness of our suggested medication compounds against the separate ccRCC causing KGs that have previously been proposed by other researchers. **Fig 1** displays the study’s procedure.

## 2. Methodology

### 2.1. Data source and descriptions

We took into account both the raw data and the meta-data related to ccRCC in order to achieve the goal of this research.

#### 2.1.1. Gene expression profile collection

Nine (9) gene expression profile datasets that are publicly available and have accession numbers GSE16441 [44], GSE53757 [45], GSE66270 [46], GSE66272 [46], GSE16449 [47], GSE76351 [48], GSE66271 [46], GSE71963 [49] and GSE36895 [50] were downloaded from the Gene Expression Omnibus (GEO) databae plugin in the National Center for Biotechnology Information (NCBI) database (https://www.ncbi.nlm.nih.gov/geo/). The details of these 9 gene expression profile datasets are given in **S1 Table in S1 File**.

#### 2.1.2. Meta-data collection

A total of 110 ccRCC-associated meta-drug agents **(S2 Table in S1 File)** were collected from the published articles [30,51–59], online database [60] and other sources [61,62] to select the potential drug agents. Additionally, we chose the top 30 publicly accessible KGs that cause ccRCC **(S3 Table in S1 File)** by the literature review [8,24,30,33,36,63–105] to verify the performance of the proposed candidate drug agents by molecular docking analysis against the other independent receptors.

### 2.2. Identification of DEGs from microarray gene expression profiles

The statistical linear models for microarray (LIMMA) data analysis [106] is a popular approach for identification of differentially expressed genes (DEGs) between case and control samples [107,108].

The LIMMA model is written as,

$$z_{g}={X\alpha }_{g}+\epsilon_{g}$$ (1)

where ***z***_g_ = (z_g1_, z_g2_,…, z_gn_)^/^ is the responses (expressions) *m*-vector of *g*th gene with *n* = *n*_1_+*n*_2_ samples (*g* = 1, 2,…, *G*), ***X*** is an *n*×2 design matrix, ***α***_g_ = (*α*_*g1*_, *α*_*g2*_) ^/^ is a 2-vector (2<*n*) of effects for ccRCC and control groups of n samples and the error vector **ϵ**_g_~ N(0, ***W***_*g*_*ϭ*_g_^2^). The gth gene is said to be equally expressed gene (EEG) if the null hypothesis (H_0_): *α*_*g1*_ *= α*_*g2*_ *= > γ*_*g*_ = (*α*_*g1*_*−α*_g2_) = 0 is true. Otherwise, it is said to be a differentially expressed gene (DEG). To test the significance of H_0_, the test statistic for LIMMA approach is defined as

$${\tilde{t}}_{g}=\frac{{\hat{\gamma }}_{g}+\gamma_{g}}{{\tilde{S}}_{g}{\sqrt{\delta }}_{g}}$$ (2)

which follows *t*-distribution under H_0_. Adjusted *P*-values based on the moderated *t*-statistic and the average of log_2_ fold-change (aLog_2_FC) values of treatment group with respect to the control group were used to select DEGs or EEGs as follows,

$${DEG}_{g}=\left\{ \begin{array}{c} DEG(Up),\;if\;adj.P.value<0.05\;and\;a{Log}_{2}\;{FC}_{g}>+1.0\\ DEG(Down),if\;adj.P.value<0.05\;and\;a{Log}_{2}\;{FC}_{g}<-1.0 \end{array}\right.$$

Where,

$${aLog}_{2}\;{FC}_{g}=\left\{ \begin{array}{l} \;\frac{1}{n_{1}}\sum_{i}^{n_{1}}{log}_{2}{(z}_{gi}^{T})-\frac{1}{n_{2}}\sum_{j}^{n_{2}}{log}_{2}{(z}_{gj}^{C}),\;if\;n_{1}\neq n_{2}\\ \frac{1}{n}\sum_{i}^{n}{log}_{2}(\frac{z_{gi}^{T}}{z_{gj}^{C}}),\;if\;n_{1}=n_{2}=n \end{array}\right.$$

Here $z_{gi}^{T}$ and $z_{gj}^{C}$ are the responses/expressions for the *g*th gene with the *i*th case and *j*th control samples, respectively. We applied the LIMMA R-package [109] for computing the *P*-values and aLog2FC values to separate the DEGs/EEGs from each of nine datasets, significantly. Then we selected the common DEGs (cDEGs) from 9 DEGs-sets as ccRCC causing genes.

### 2.3. Screening of cDEGs through survival analysis

To select the cDEGs having significant prognostic power, we performed survival analysis by using the GEPIA2 [110] web-tool linked with TCGA and GTEx databases. The GEPIA web-tool performed survival analysis by using Next-Generation Sequencing (NGS) based RNA-seq profile datasets from both TCGA and GTEx databases. We constructed two Kaplan-Meier survival probability curves for each of cDEGs based on its low and high expression groups. The significant difference between two curves was assessed by using log-rank test with *P*-value <0.1. The significant difference between two survival curves indicates the strong prognostic performance of the gene. The reduced set of cDEGs-set based on survival analysis was denoted as scDEGs for convenience of presentation.

### 2.4. Selection of key genes (KGs) from scDEGs

Proteins interact with one another within cells to carry out their functions, and the protein-protein interaction (PPI) network’s information helps us understand how proteins operate better [111]. A PPI network of DEGs was created using the web database and analysis tool "Search Tool for the Retrieval of Interacting Genes" (STRING v11) [38]. The STRING web tool produces information based on anticipated and experimental interactions as well as 3D structures, additional data, and a confidence score [112]. To improve the network’s visual representation, the acquired PPIs are analyzed using Cytoscape [113]. Eleven topological measures rendered available by the CytoHubba plugin for Cytoscape can be utilized to rank the PPI network nodes utilized for KG identification [114]. Degree, MNC, Maximal Clique Centrality (MCC), Bottleneck, Closeness, Betweenness, and Edge Percolated Component (EPC) were the seven topological measures that were taken into account in this research. Based on several topological evaluations of the PPI-network, the greatest percentage of interactions was used to pick the highest-ranked KGs.

### 2.5. Functional enrichment analysis of scDEGs highlighting KGs

Functional enrichment analysis of a gene-set identifies biological functions or pathways that are overrepresented among a set of genes compared to a background. This process reveals significant associations between genes and biological terms to understand the roles of those genes in a specific biological term (function/processes/pathways). The scDEGs-set enrichment analysis with three (3) Gene Ontology (GO) terms Biological Process (BP), Molecular Function (MF), and Cellular Component (CC), were conducted in order to comprehend cellular function, the molecular activity, and location inside the cell where scDEGs including KGs perform their tasks [40]. A typical tool for understanding metabolic pathways is the "Kyoto Encyclopedia of Genes and Genomes" (KEGG) pathway, which utilizes extensively gene annotation [41]. The DAVID web server was used to analyze GO terms and KEGG pathways to understand the biological importance of scDEGs, with a particular focus on key genes [115]. In this instance, the threshold was set to the adjusted *P*-value < 0.05 for selecting the significantly enriched functions by the Fisher exact test.

### 2.6. Pan-cancer analysis of KGs

Pan-cancer analysis was performed to verify the association of KGs with ccRCC including different other cancers based on independent database. Using the TCGA database [37] and the TIMER2.0 (Tumor Immune Estimation Resource) web-tool [39], we conducted a pan-cancer study for each of KGs to look into its potential function in pan-cancer. The significant difference between two groups for each cancer was examined by Wilcoxon signed rank test with the cutoff at *P*-value <0.1.

### 2.7. Association of KGs with different immune infiltration levels in ccRCC

Immune infiltration analysis with KGs investigates how their expression levels are statistically associated with different immune cell types and their abundance within a tissue or tumor. This analysis helps to understand how KGs and immune cell infiltration stimulate each other, providing insights into the interactions between gene expression and immune responses in the disease context. The Tumor Immune Estimation Resource (TIMER 2.0) [39] is a comprehensive tool that estimates the quantity of tumor-infiltrating immune cell types from TCGA data. We utilized TIMER’s online tools to investigate the immune infiltration levels of CD8+ T cells, CD4+ T cells, neutrophils, B cells, macrophages, and dendritic cells with KGs in ccRCC.

### 2.8. DNA methylation of KGs

DNA methylation of KGs stimulates their expressions and regulations by adding methyl groups to their DNA. It helps to identify significant changes in gene activity associated with diseases, providing insights into gene regulation mechanisms and contributing to understanding disease processes [116]. MethSurv web-tools with TCGA-KIRC methylation data were used to investigate DNA methylation, a complex epigenetic process that controls gene expression in both normal and malignant cells [42]. DNA methylation values (ranging from 0 to 1) were represented by β values, which were computed as M/(M + U + 100) for every CpG site. The intensities of methylation and unmethylation are represented by M and U, respectively. We classified the methylation levels into two groups based on the difference in methylation β value between the cut-off point and higher (methylation β value above) in order to assess the impact on patient survival. One can use data quantiles or the means to get the grouping cut-off point.

### 2.9. Detection of top regulators of KGs

Transcription factors (TFs) and microRNAs (miRNAs) are considered as the transcriptional and post-transcriptional regulators of protein coding genes. The regulatory network analysis was conducted to explore TFs-proteins and miRNAs as the transcriptional and post-transcriptional regulators of KGs. The network of TFs and KGs links were examined by using the JASPAR database [117] in order to pinpoint the primary TFs associated with KGs. By using the TarBase database [118] to examine miRNA-KG connections, we identified key miRNAs that impact KGs at the post-transcriptional level. NetworkAnalyst [119] was used to replicate these relationships. The post-transcriptional regulators of KGs were selected from the top-ranked miRNAs. The Cytoscape tool [113] was used to show the networks of their interactions.

### 2.10. KGs-guided drug repurposing

There are two main in-silico methods for drug discovery: de-novo design, which is time-consuming and costly, and drug repurposing (DR), which utilizes existing, approved drugs for new diseases. Both methods use molecular docking to evaluate potential drug candidates, by computing their binding affinities and interactions with target proteins. Molecular docking analysis is a popular approach for exploring potential drug-molecules from huge number of alternatives to inhibit disease-causing genes [120,121]. Therefore, in this study, we also used this analysis to investigate KGs-guided potential therapeutic molecules/ligands for the treatment of ccRCC. For the purposes of docking study, we considered KGs-mediated receptor proteins and the regulatory TFs proteins that accompany them. The Protein Data Bank (PDB) [122], AlphaFold Protein Structure Database [123], and SWISS-MODEL [124] were the sources from which the three-dimensional (3D) receptor structures were obtained. The 3D structures of possible medicinal agents were obtained by searching the PubChem database [125]. The 3D architectures of protein interactions were visualized using the Discovery Studio Visualizer [126]. AutoDock tools were used to pre-process the receptor proteins, which involved removing water molecules and adding charges [127]. The Avogadro [128] was used to reduce the energy of the drugs agents, and AutoDock tools [127] were used to preprocess them. After that, AutoDock Vina was used to perform molecular docking between receptors and ligands in order to determine their binding affinity scores (kcal/mol) [129]. Let B_*mn*_ denote the binding affinity score between *m*^th^ receptors (*m =* 1, 2, …, q) and *n*^th^ ligands/agents (*n =* 1, 2,…, p). Receptors were sorted based on the descending order of their average scores $\left(\frac{1}{q}\sum_{n=1}^{p}B_{mn},\;m=1,2\ldots q\right)$, while ligands/agents were ranked by the descending order of their average scores $\left(\frac{1}{p}\sum_{m=1}^{q}B_{mn},\;n=1,2,\ldots ,p\right)$. This process was used to select the top-ranked ligands/agents as potential drug candidates.

### 2.11. ADME/T analysis of top-ranked drug molecules

ADME/T analysis assesses a drug candidate’s absorption, distribution, metabolism, excretion, and toxicity to predict its safety and effectiveness. In drug repurposing, a compound may show strong binding in docking studies with a new target, suggesting potential for a new use. However, if it fails ADMET criteria—like poor absorption, rapid metabolism, or high toxicity—it is unlikely to succeed in this new role. ADMET analysis helps filter out such candidates, ensuring that only those with favorable pharmacokinetics and safety profiles move forward in the repurposing process. We analyzed the drug-like properties and ADME/T (absorption, distribution, metabolism, excretion, and toxicity) profiles of the top ten ranked drug compounds to better understand their structural features and chemical descriptors. The SCFBio web application [130] was used to evaluate compliance with Lipinski’s rule. ADME/T parameters were then predicted using the online databases SwissADME [131] and pkCSM [132], utilizing the optimal structures of the drug compounds in SMILES format for the calculations.

## 3. Results

### 3.1. Identification of DEGs by LIMMA approach

We have identified the genes that are differentially expressed (DEGs) in each of the nine datasets comparing ccRCC and control samples by applying the statistical LIMMA technique. Adj.*P*.value ≤ 0.05 and log_2_FC > 1 for upregulated DEGs and adj.*P*.value ≤ 0.05 and logFC < -1 for downregulated DEGs were the cutoff values, as stated in section 2.2. From nine datasets with NCBI accession IDs GSE16441, GSE53757, GSE66270, GSE66272, GSE8449, GSE76351, GSE66271, GSE71963 and GSE36895, we identified 413, 1602, 2430, 2929, 1448, 899, 2748, 314, 1160 upregulated DEGs and 1168, 352, 1294, 1584, 1378, 694, 2473, 496, 1980 downregulated DEGs, respectively. The total number of cDEGs was found 133; of these, 26 were downregulated and the remaining 107 were upregulated (**S4 Table in S1 File**).

### 3.2 Screening cDEGs through survival analysis

We selected 54 cDEGs out of 133 based on the significant difference (*P*-value ≤0.1) between two survival probability curves with the low and high expression groups in the independent TCGA and GTEx databases (**S5 Table in S1 File**). The reduced cDEGs-set based on survival analysis was denoted as scDEGs for convenience of presentation.

### 3.3. Identification of Key Genes (KGs) from scDEGs

**Fig 2:**
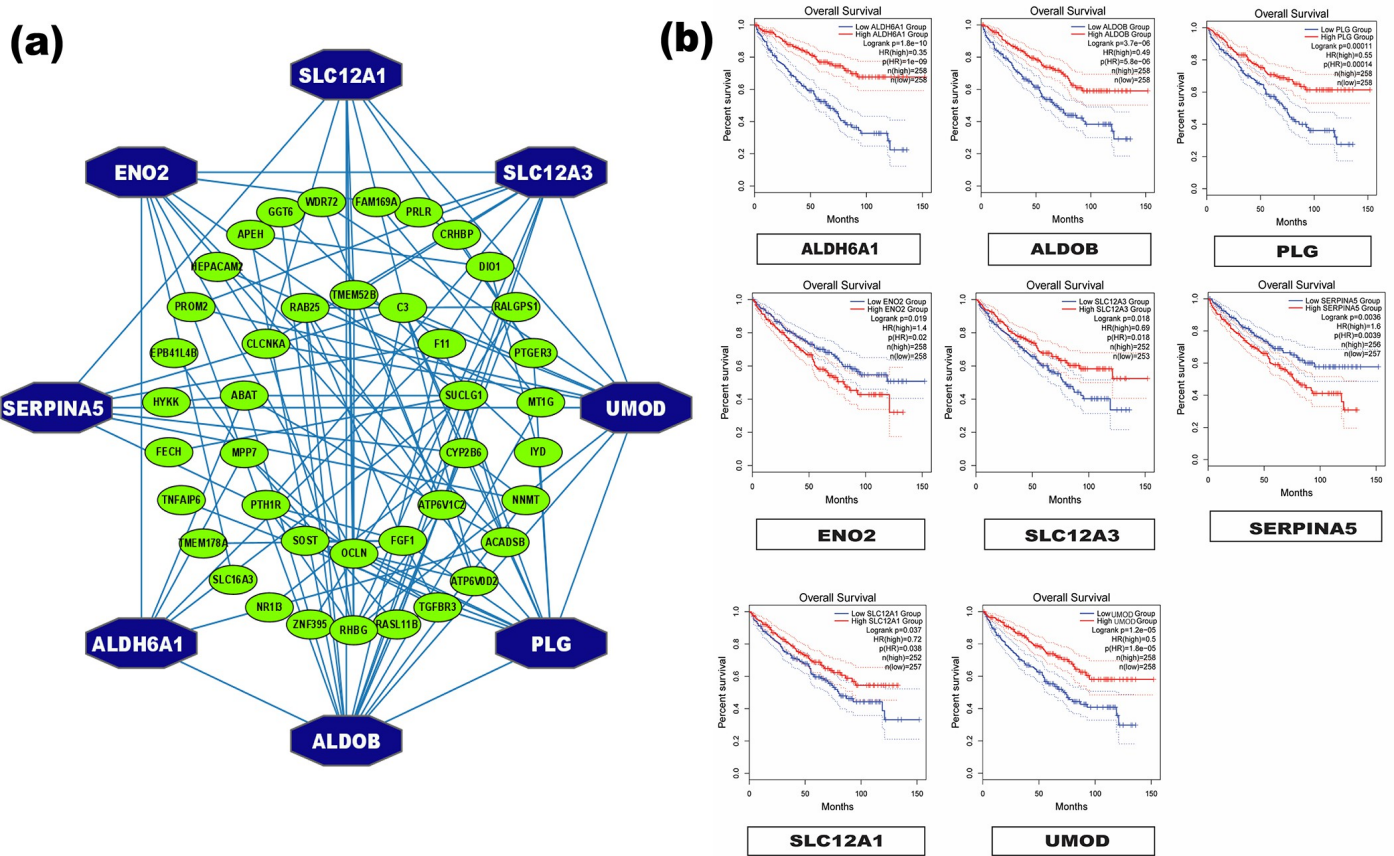
(a) Identification of KGs by the PPI network analysis of scDEGs, Where Hexagonal nodes in blue colour represent the KGs, (b) Survival probability curves with the low and high expression groups with KGs.

A PPI network with 113 edges and 54 nodes for 54 scDEGs was built. Using seven topological measures (Degree, MNC, MCC, EPC, Closeness, Betweenness and BottleNeck) with the PPI network, we detected the top eight KGs that are *PLG*, *ENO2*, *ALDOB*, *UMOD*, *ALDH6A1*, *SLC12A3*, *SLC12A1*, *and SERPINA5* (**Fig 2 and S6 Table in S1 File**).

### 3.4. Functional analysis of scDEGs highlighting KGs

To investigate the KG-involved pathogenetic processes of ccRCC, we conducted GO and KEGG pathway enrichment analysis on 133 scDEGs **(Table 1)**. In order to highlight the KGs, the top five keywords from each of the KEGG pathways, cellular components, molecular functions and biological processes (CC, MF and BP) were considered in this work. potassium ion homeostasis, glycolytic process, chloride ion homeostasis, response to xenobiotic stimulus, chloride transmembrane transport were the top 5 highly enriched BPs; CCs were apical plasma membrane, extracellular exosome, mitochondrial matrix, extracellular region, plasma membrane; MFs were heparin binding sodium:potassium:chloride symporter activity, cation: chloride symporter activity, potassium: chloride symporter activity, identical protein binding, and KEGG pathways were Metabolic pathways, Complement and coagulation cascades, Propanoate metabolism and Carbon metabolism.

**Table 1:** Significantly enhanced KEGG pathways and GO function with cDEGs through the involvement of KGs linked to the pathogenetic processes of ccRCC.

**Biological processes (BPs)**				
**GO ID**	**GO function**	**No of scDEGs**	***P*.Value**	**Associated KGs**
GO:0055075	potassium ion homeostasis	10	0.001	SLC12A 3, SLC12A1, UMOD
GO:0006096	glycolytic process	9	0.010	ALDOB, ENO2, ALDH6A1
GO:0055064	chloride ion homeostasis	5	0.028	SLC12A3, ALDH6A1
GO:0009410	response to xenobiotic stimulus	9	0.029	UMOD, ENO2
GO:1902476	chloride transmembrane transport	13	0.030	SLC12A1, SERPINA5
**Cellular Components (CC)**				
**GO ID**	**GO function**	**No of scDEGs**	***P*.Value**	**Associated KGs**
GO:0016324	apical plasma membrane	8	4.37E-05	SLC12A3, SLC12A1, UMOD
GO:0070062	extracellular exosome	17	7.36E-05	SLC12A3, SLC12A1, UMOD, PLG, ENO2, SERPINA5, ALDOB
GO:0005759	mitochondrial matrix	6	0.003	ALDH6A1, PLG, ENO2
GO:0005576	extracellular region	12	0.015	SERPINA5, SERPINA5, ALDOB,
GO:0005886	plasma membrane	21	0.031	SLC12A3, SLC12A1, PLG,ENO2
**Molecular Functions (MFs)**				
**GO ID**	**GO function**	**No of scDEGs**	***P*.Value**	**Associated KGs**
GO:0008201	heparin binding	5	0.001	SERPINA5, UMOD, PLG, ENO2
GO:0008511	sodium:potassium:chloride symporter activity	8	0.006	SLC12A3, SLC12A1, PLG, ENO2
GO:0015377	cation: chloride symporter activity	4	0.009	SLC12A3, SLC12A1, PLG, ENO2
GO:0015379	potassium: chloride symporter activity	4	0.020	SLC12A3, SLC12A1, ALDH6A1, ENO2
GO:0042802	identical protein binding	9	0.055	ALDOB, ENO2
**KEGG Pathways**				
**KEGG ID**	**KEGG function**	**No of scDEGs**	***P*.Value**	**Associated KGs**
hsa01100	Metabolic pathways	13	0.002	ENO2, ALDH6A1
hsa04610	Complement and coagulation cascades	6	0.003	PLG, SERPINA5
hsa00640	Propanoate metabolism	4	0.005	ALDH6A1, SLC12A3, SLC12A1, PLG
hsa01200	Carbon metabolism	9	0.006	ALDH6A1, ALDOB, ENO2

### 3.5. Pan-cancer analysis of KGs

**Fig 3:**
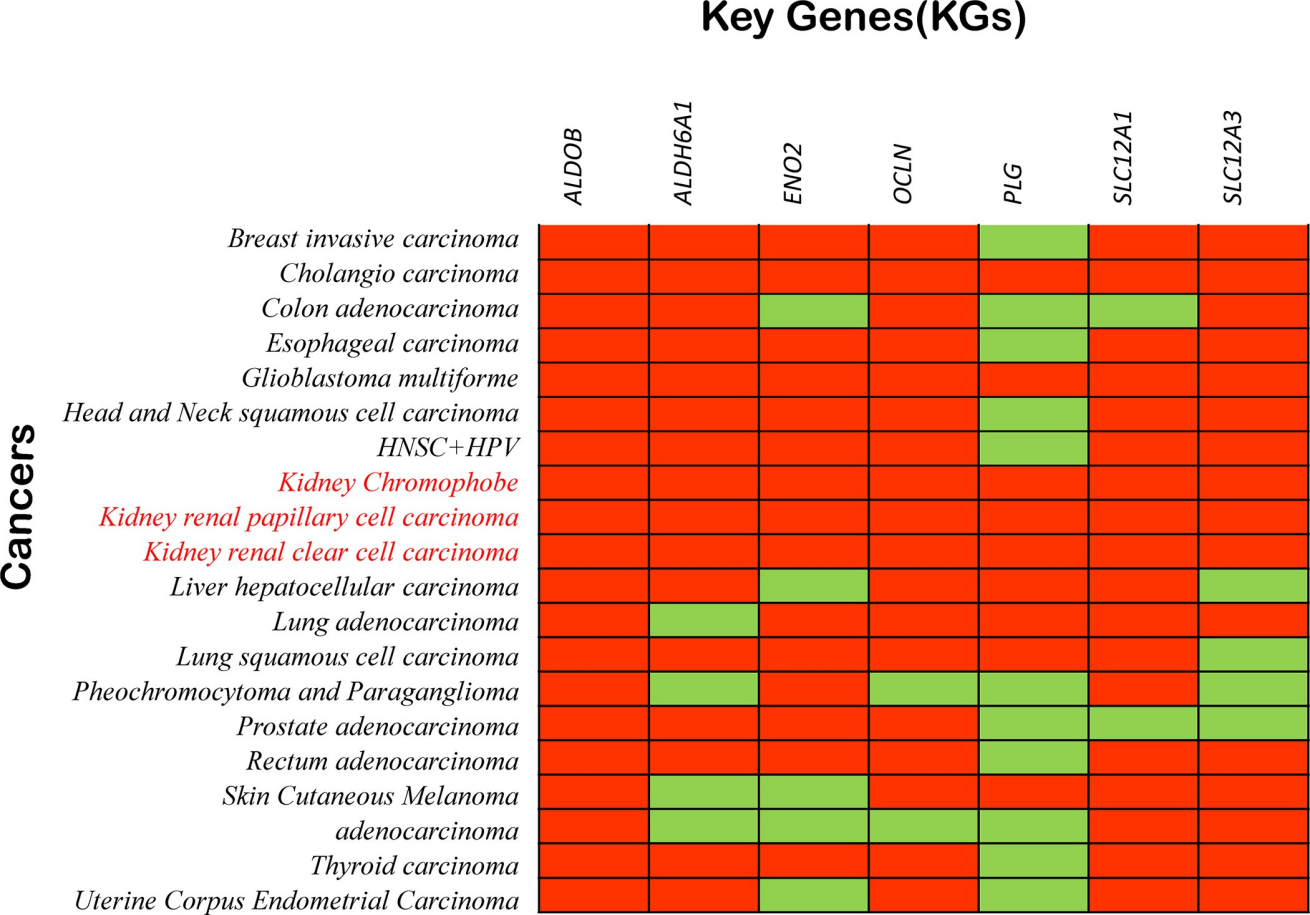
Using the TCGA database and the TIMER2 online tool, pan-cancer analysis was used to identify the top 20 cancers associated with KGs. Orange and green colors, respectively, indicate significant (*P*-value <0.01) and insignificant (*P*-value ≥0.01) pairwise gene-disease relationships.

To examine the potential function of KGs in pan-cancer, we also conducted a pan-cancer study using the TCGA database, focusing on the top 20 malignancies, as seen in **Fig 3**. The kidney chromophobe, kidney renal papillary cell carcinoma, and kidney renal cear cell carcinoma are the three tumors that we demonstrated in this figure to be substantially correlated with KGs **(S8 Table in S1 File)**.

### 3.6. Immune infiltration level analysis of KGs in ccRCC

The tumor microenvironment (TME) is a complex environment composed of different stromal components including immune cell along with the tumor cells [133–135]. Immune infiltration analysis involves assessing the types, densities, and functional states of immune cells within the tumor microenvironment to understand their impact on cancer progression and therapeutic responses, particularly in the context of immunotherapy efficacy [136–139]. RCC is one of the tumors with the highest immune infiltration rate [140–142]. The Tumor Immune Estimation Resource (TIMER 2.0) [39] is a comprehensive tool that estimates the quantity of tumor-infiltrating immune cell types from TCGA data. To predict the infiltration of immune cells in ccRCC by the TIMER algorithm, we assessed the correlations between the expression levels of the KGs and the levels of infiltration of six immune cells (CD8+ T cell, B cell, CD4+ T cell, dendritic cell, neutrophil, and macrophage) (**S1 Fig in S1 File**). These findings indicate that the majority of tumor-infiltrating immune cells in ccRCC, with the exception of CD4+ T(0.11≥ Rho ≥ 0.28) cells, have a negative correlation with KGs (CD8+ T cell (-0.20 ≤ Rho ≤ 0.32), B cell (-0.11≤ Rho ≤ 0.24), Macrophage (-0.23≤ Rho ≤ 0.21), Neutrophil (-0.14 ≤ Rho ≤ 0.29)), indicating a special relationship between tumor metabolism and immunological infiltration. This result could help to discover potential immunotherapy for ccRCC. These findings strongly suggest that KGs plays an important role in immune infiltration in ccRCC.

### 3.7. DNA methylation of KGs

The development of cancer may be significantly influenced by DNA methylation at several CpG (CG) sites in KGs. Thus, we used the TCGA-KIRC methylation data and the MethSurv online tool to analyze DNA methylation at CpG sites for KGs (*PLG*, *ALDH6A1*, *ENO2*, *ALDOB*, *SERPINA5*, *SLC12A1*, *SLC12A3*, *and UMOD*). **Table 2** shows that all KGs were notably (*P-*value of <0.01) within 5 KGs (*ENO2*, *SERPINA5*, *SLC12A1*, *SLC12A3*, *UMOD*) were hypomethylated and 3 KGs (*PLG*, *ALDH6A1*, *ALDOB*) were hypermethylated at various CpG locations.

**Table 2:** The significant prognostic value of CpG in KGs.

KGs	Genomic Region	Relation to CpG island	CpG site	HR	LR test (*P-*Value)
PLG	TSS1500	Open_Sea	cg04181478	2.109	0.003596
PLG	5’UTR	Open_Sea	cg08531365	2.834	3.22E-07
ALDH6A1	Body	N_Shelf	cg14849468	3.099	4.07E-05
ENO2	TSS200	Island	cg02531908	0.424	0.000725
ENO2	TSS200	Island	cg22977254	0.488	0.001793
ALDOB	TSS1500	Open_Sea	cg13585586	2.742	0.000193
ALDOB	5’UTR	Open_Sea	cg14316227	2.742	0.000312
SERPINA5	5’UTR	Open_Sea	cg16937611	0.462	0.003249
SERPINA5	Body	Open_Sea	cg18731460	1.934	0.001429
SLC12A1	TSS1500	Open_Sea	cg07915221	0.442	0.000104
SLC12A1	Body	Open_Sea	cg14930674	0.422	1.18E-05
SLC12A1	Body	Open_Sea	cg23530596	3.148	3.19E-05
SLC12A3	TSS200	Open_Sea	cg01191774	0.295	9.22E-06
SLC12A3	1stExon	Open_Sea	cg03455024	0.432	0.001318
SLC12A3	TSS200	Open_Sea	cg04303879	0.46	0.001954
UMOD	Body	S_Shore	cg03084308	0.292	7.18E-06
UMOD	Body	Island	cg06861044	3.327	4.78E-05
UMOD	5’UTR	S_Shelf	cg07456201	0.393	0.000291
UMOD	3’UTR	Open_Sea	cg07817806	2.062	0.006039
UMOD	Body	N_Shelf	cg27605307	0.431	0.001249874

### 3.8. Identification of key regulators for KGs

**Fig 4:**
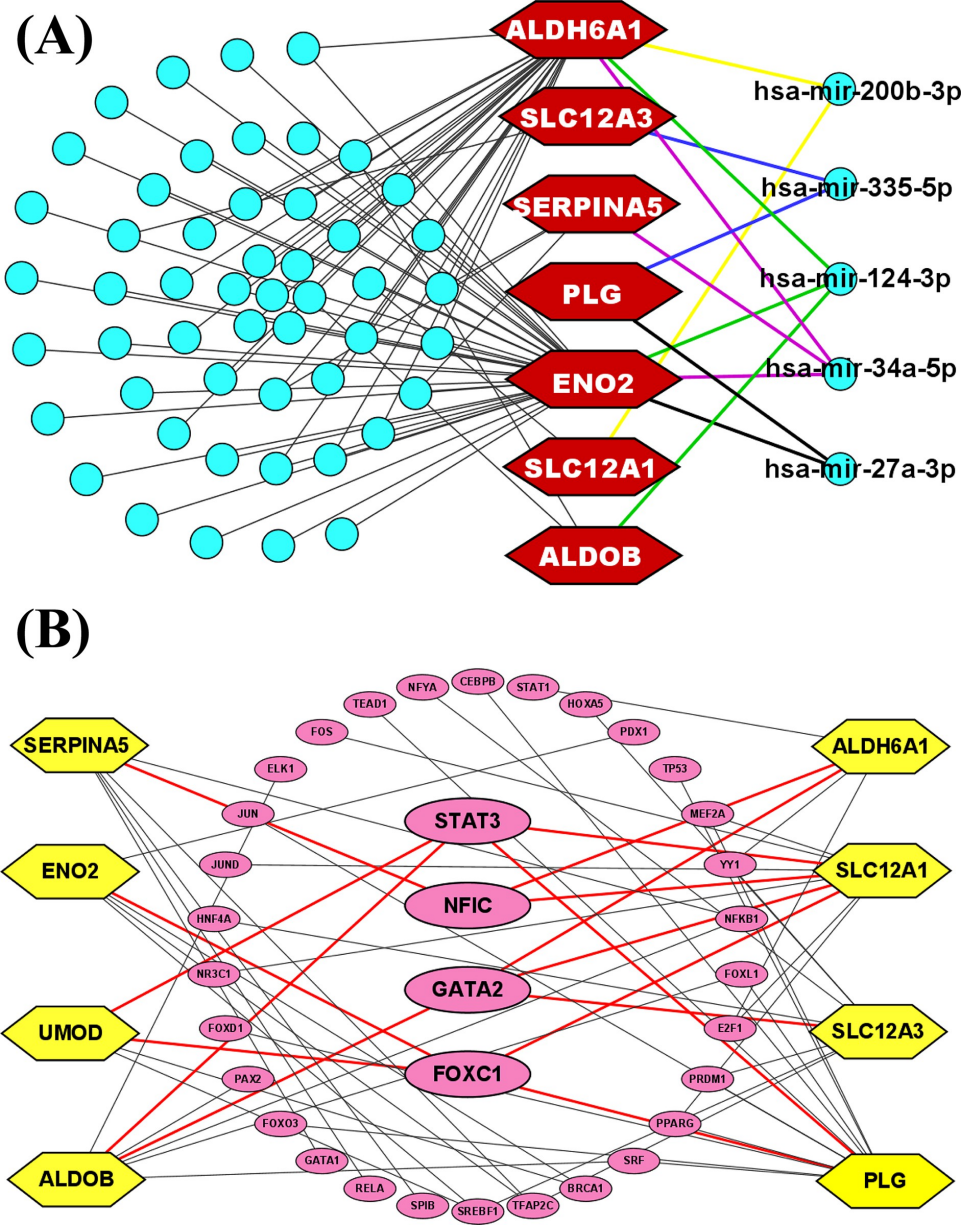
(A) miRNA-KGs interaction network with. Here, KGs were denoted by hexagon-shaped markings in the color red. The top miRNAs were denoted by circles with a blue color. (B) TF-KGs interaction network. Here, KGs were denoted with yellow hexagon shapes and the top -TFs denoted with pink circles.

We investigated the networks of TFs and miRNAs with KGs to identify the transcriptional and post-transcriptional regulators of the KGs. Initially, we chose the top four transcription factors (TFs), namely GATA2, FOXC1, NFIC, and STAT3, to serve as the transcriptional regulators of KGs. This selection was based on two key topological metrics: betweenness and degree, where the cutoff values were set at ≥75.88 and ≥3, respectively **(Fig 4A)**. Then, applying the same topological metrics with cutoff degree ≥2 and betweenness ≥120, respectively, we chose the top five miRNAs (hsa-mir-124-3p, hsa-mir-34a-5p, hsa-mir-27a-3p, hsa-mir-335-5p, and hsa-mir-200b-3p) as the post-transcriptional regulators KGs **(Fig 4B)**.

### 3.9. Exploring KGs-guided candidate drugs by molecular docking

**Fig 5:**
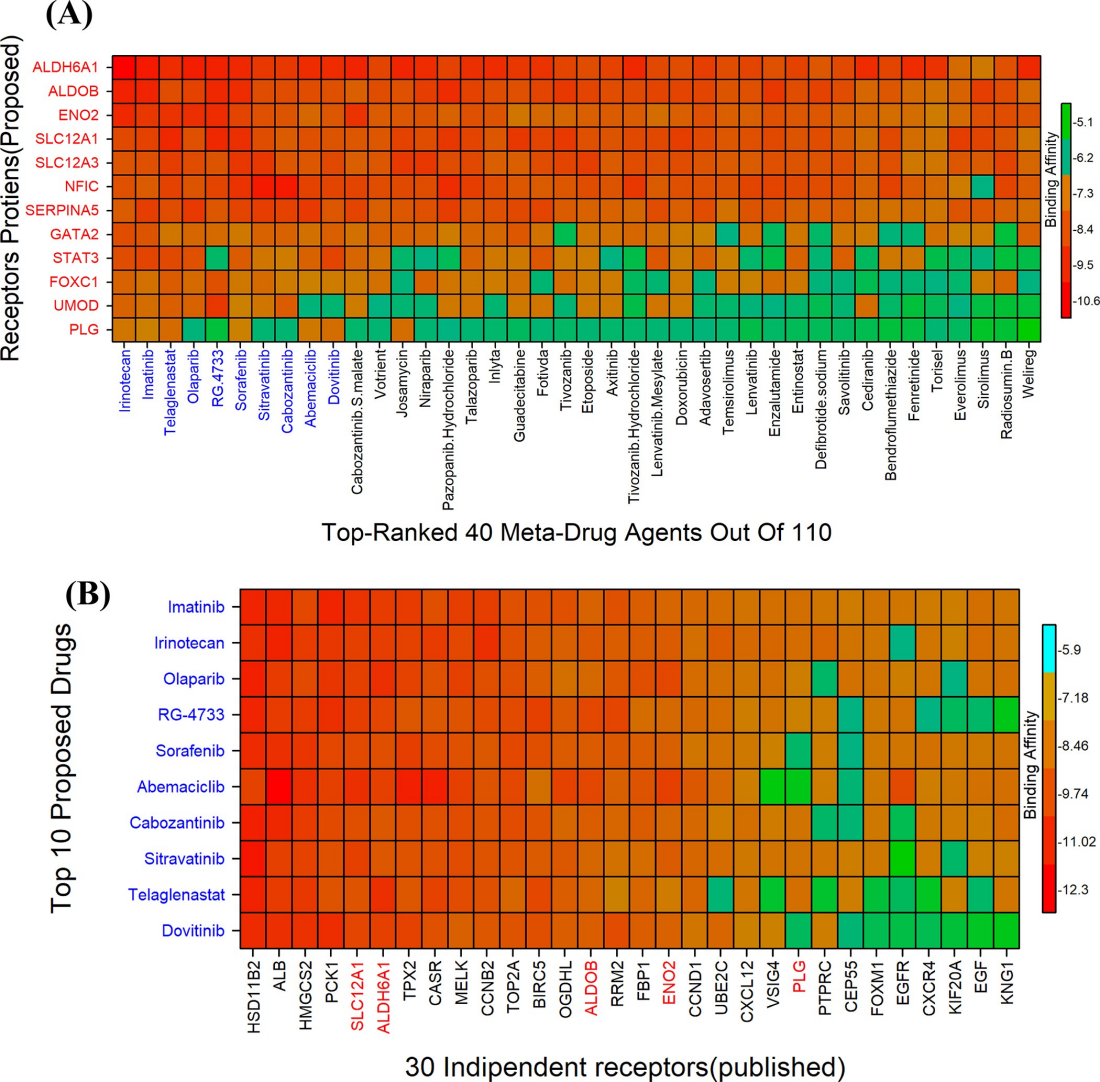
Image of drug-target binding affinity matrices (A) X-axis indicates top-ordered 40 drug agents (out of 110) and Y-axis indicates ordered proposed receptor proteins. (B) the top 10 proposed and published drugs (out of 111) in the Y-axis and the top 30 target proteins (published by others) in the X-axis.

As the m = 12 drug target receptors, we employed 8 KGs and the 4 TFs that govern them. The three-dimensional (3D) structures of eight of them (ALDOB, ENO2, OCLN, PLG, SERPINA5, STAT3, GATA2, UMOD) were obtained from Protein Data Bank (PDB) by using the PBD codes 8d44, 2akz, 1xaw, 1b2i, 2ol2, 5u5s, 5o9b, and 6zs5). The 3D-structures of ALDH6A1, FOXC1, SLC12A1, and SLC12A3 were obtained using the "AlphaFold Protein Structure Database" using UniProt with ID Q02252, Q12948, Q13621 and P55017. Uniport ID P08651 was used to obtain the 3D structures of the rest targets (NFIC) from SWISS-MODEL. Molecular docking study between our potential receptors and meta-drug compounds was used to compute the binding affinity scores (BAS). Subsequently, we choose a limited set of drugs as potential drugs by arranging the target receptors according to their row sums and column sums in the binding affinity matrix. A = (X*ij*) (**Fig 5A & S8 Table in S1 File)**. All 13 receptor proteins had strong bindings with the top four lead drugs, irinotecan, imatinib, telaglenastat, and RG-4733 (BAS< - 7.0 kcal/mol). Then, 12 of the 13 receptor proteins had strong bonds with sorafenib, nexavar, sitravatinib, dactinomycin, olaparib, and capozantinib (BAS < - 7.0 kcal/mol). Consequently, we examined those ten lead drugs as possible ccRCC treatments. To assess the potential of the indicated candidate drugs to bind to the most sophisticated alternative independent receptors, we looked at 48 papers that postulated KGs that may induce ccRCC. ALB, ALDH6A1, ALDOB, BIRC5, CASR, CCNB2, CCND1,CEP55, CXCL12, CXCR4, EGF,EGFR, ENO2, FBP1, FOXM1,HMGCS2, HSD11B1, KIF20A, KNG1, MELK, OGDHL, PCK1,PLG, PTPRC,RRM2, SLC12A1, TOP2A,TPX2, UBE2C, VSIG4 were published in at least three studies were taken into consideration **(S3 Table in S1 File)**. These 30 KGs-induced proteins were thought to be distinct receptors. The 3D structures of ALB, ALDOB, BIRC5, CASR, CCND1, CEP55, CXCL12, CXCR4, EGF, EGFR, ENO2, FBPI, FOXM1, HMGCS2, KIF20A, KNG1, MELK, PCK1, PLG, PTPRC, RRM2, TOP2A, TOX2, UBE2C, and VSIG4 were obtained using the codes 1e7a, 2akz, 1xox, 5k5t, 6p8e, 3e1r, 1sdf, 2n55, 1p9j, 1z9i, 1b2i, 1fta, 7fj2, 2wya, 6yip, 6f3v, 4d2p, 1M51, 1b2i 5fn7, 30lj, 1zxm, 6vph, 1i7k, and 2icc, respectively. The UniProt IDs Q02252, O95067, P80365, Q9ULD0, and P55017 allowed researchers to get the three-dimensional structures of ALDH6A1, CCNB2, HSD11B1, OGDHL, and SLC12A1 from the "AlphaFold Protein Structure Database". We found that our top 10 proposed drug agents can also effectively bind to 30 independent receptors. Nearly all of these receptors demonstrated strong binding affinities with these agents **(Fig 5B and S9 Table in S1 File).** As a result, we heartily advise that the ten drugs that have been suggested (Irinotecan, Imatinib, Telaglenastat, Olaparib, RG-4733, Sorafenib, Sitravatinib, Cabozantinib, Abemaciclib, and Dovitinib) may be better options for treating ccRCC. **S10 Table in S1 File** displays the 3D interactions between therapeutic compounds and the top three likely receptors.

### 3.10. Drug likeness, ADME and toxicity analysis

The ADME properties of a drug molecule are employed to evaluate how it is absorbed, distributed, metabolized, excreted, and its potential toxicity. Conversely, drug-likeness properties analyze the molecule’s physicochemical characteristics to understand its diverse chemical properties (**Table 3**). According to Lipinski’s rule, these ten (Irinotecan, Imatinib, Telaglenastat, Olaparib, RG-4733, Sorafenib, Sitravatinib, Cabozantinib, Abemaciclib, and Dovitinib) drug molecules were classified as drugs as they satisfied at least four of Lipinski’s five requirements **(S11 Table in S1 File)**. The standard range (1 to less than equal 5) [130] of Lipinski’s rule is supported by the lipophilicity (LogP value) of these 10 medications. Based on a comparison of their LogP values with standard values, these substances are identified as lipophilic. Consequently, almost all of the drug-likeness criteria has been met by our proposed top ten drug compounds (**S11 Table in S1 File**). To assess the efficacy and indemnity of the suggested drugs, many parameters can be used to analyze their toxicity and ADME analyses. The chemicals are a possible oral medication candidate since it is expected that they would be sufficiently absorbed in the gastrointestinal tract. In the human intestines, a chemical is considered well absorbed if its Human Intestinal Absorption (HIA) score is > 30% [143,144]. Our study revealed that, of the ten medications we suggested, all had a high HIA score of ≥68%, indicating strong absorption characteristics by the human body. Additionally, our top eight pharmaceuticals, with the exception of RG-4733 and dovitinib, were able to block the P-glycoprotein inhibitor (P-gpI). The blood-brain barrier (BBB) permeability index calculates a compound’s capacity to pass the BBB. When a compound’s LogBB value is less than -1, it is thought to be poorly disseminated over the BB barrier, but compounds with a LogBB value of 0.3 or above can possibly pass the BBB. After evaluating all substances, it was found that none of the suggested medications could effectively cross the blood-brain barrier, as all had BBB values below 0.3 (**Table 3**). Additionally, they are thought to partially reach the central nervous system based on the LogPS (CNS) value. Human cytochrome P 450 (CYP) enzymes are hemoproteins that are membrane-bound and are crucial to homeostasis, drug detoxification, and cellular metabolism. More than one CYP from CYP classes 1–3 is responsible for over 80% of the oxidative metabolism and around 50% of all common clinical drug elimination in humans [145]. All medications possess the YES characteristics necessary to block the human body’s CYP3A4 membrane. A drug’s toxicity is proportional to its LC50 value; a smaller number denotes more toxicity (<1.0 log mM). All of our proposed drugs had LC50 value greater than 1.0 log mM. The toxicity analyses of our proposed compounds evaluating AMES tests, fatal dose LD50, and minnow toxicity LC50 indicated that they were inert across all criteria. Consequently, they are predicted to be non-toxic, displaying drug-like properties and suitability for oral consumption.

**Table 3:** ADME/T profile of top-ranked ten drugs.

Compounds	Absorption			Desorption		Metabolism	Excretion	Toxicity		
	Caco2 Permeability	HIA (%)	P-gpI	BBB	CNS	CYP3A4	TC	AMES	LC_50_ (log mM)	LD_50_ (mole/kg)
				(Permeability)						
**Imatinib**	1.09	93.84	Yes	-1.37	-2.51	Yes	0.71	No	2.08	2.9
**Irinotecan**	0.64	99.87	Yes	-1.30	-3.23	Yes	0.93	No	1.78	2.81
**Telaglenastat**	0.77	80.76	Yes	-1.15	-3.67	Yes	0.63	No	2.32	2.13
**Olaparib**	1.08	91.92	Yes	-0.85	-2.65	Yes	0.56	No	1.96	2.62
**RG-4733**	-0.59	68.00	No	-1.28	-3.12	Yes	0.97	No	2.83	2.48
**Sorafenib**	0.76	85.49	Yes	-1.47	-2.02	Yes	0.61	No	1.51	2.14
**Sitravatinib**	0.34	99.71	Yes	-1.92	-3.37	Yes	2.09	No	1.94	3.11
**Cabozantinib**	0.16	100	Yes	-0.67	-3.04	Yes	0.75	No	3.37	2.44
**Abemaciclib**	1.53	83.94	Yes	-1.57	-3.21	Yes	0.58	No	1.78	2.37
**Dovitinib**	0.47	83.63	No	-0.70	-2.26	Yes	0.75	No	3.03	2.40

## 4. Discussion

**Fig 6:**
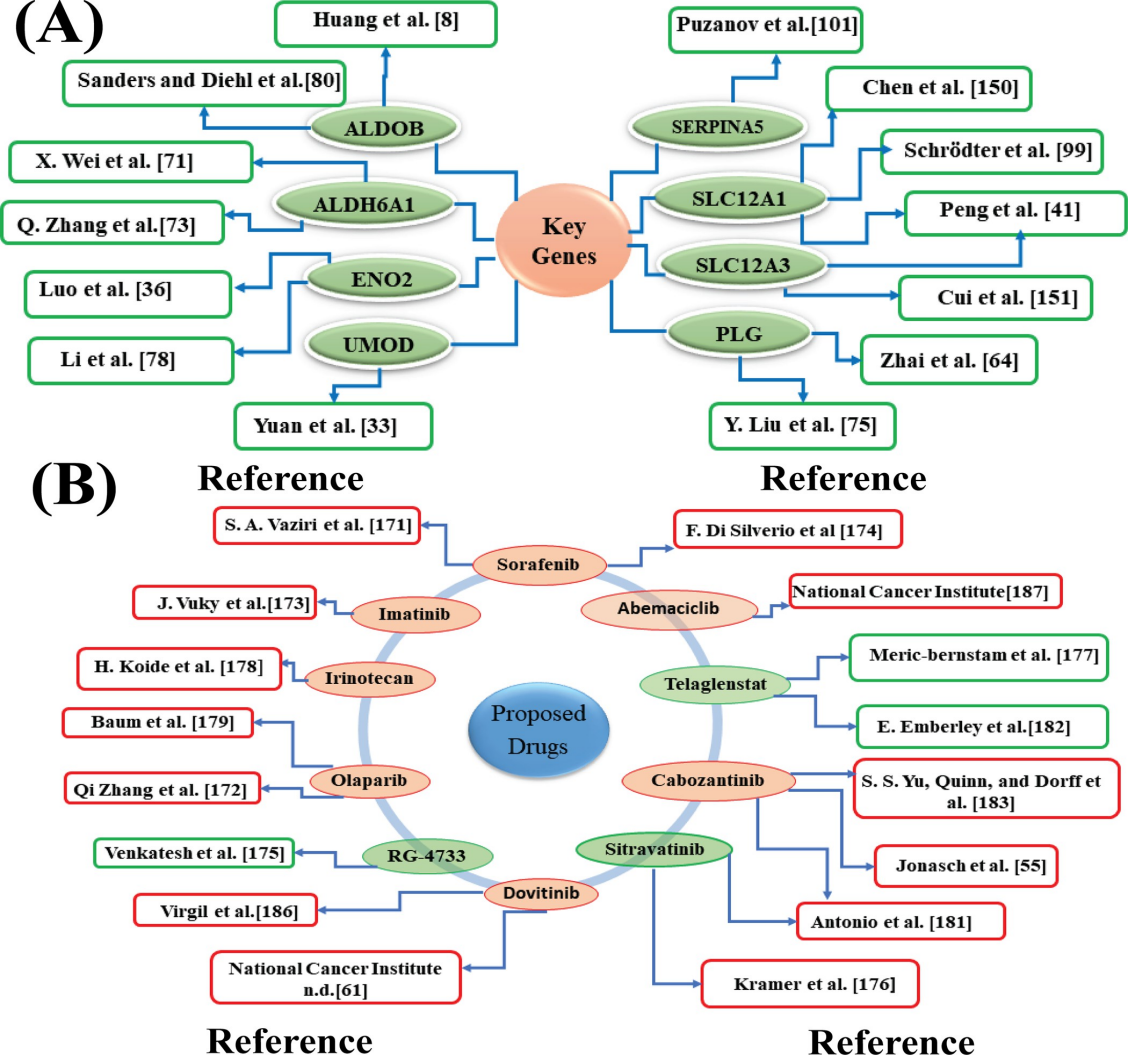
Supporting articles for the proposed ccRCC-causing KGs and suggested drug molecules. (A) Supporting articles with the KGs and (B) supporting articles with the suggested drugs, where the red ellipse indicates FDA-approved drugs and the experimental drugs are indicated by the green ellipse.

RCC, is often considered as a metabolic disease, especially ccRCC. It’s target gene mutations associated with metabolic pathways are a clear characteristic of RCC [146–149]. To investigate the genetic mechanism of ccRCC, we identified ccRCC-causing key genes (KGs) highlighting their pathogenetic processes. In order to explore ccRCC-causing KGs, we analyzed nine microarray gene-expression datasets (GSE16441, GSE53757, GSE66270, GSE66272, GSE16449, GSE76351, GSE66271, GSE71963 and GSE36895). To analyze the datasets, we first used the statistical LIMMA technique for finding the DEGs between ccRCC and control samples from each of the nine datasets. 133 common DEGs (cDEGs) from nine DEGs-sets were identified. Then, we selected top-ranked 54 cDEGs by the screening of cDEGs through the survival analysis. For convenience of presentation, we denoted this reduced cDEGs-set as the screened cDEGs (scDEGs). Then the PPI network of scDEGs identified top-ranked eight scDEGs (*PLG*, *ENO2*, *ALDOB*, *UMOD*, *ALDH6A1*, *SLC12A3*, *SLC12A1*, *SERPINA5*) as the ccRCC-causing KGs **(Fig 2).** The pan-cancer analysis also significantly supported their association with ccRCC. Additionally, several independent research have supported these KGs as the ccRCC causing KGs [8,33,36,64,71,73,75,78,80,99–101,150,151] as displayed in **Fig 6.**

*ALDOB*, a key enzyme in *glycolysis* (**Table 1**), facilitates the breakdown of fructose-1,6-bisphosphate into glyceraldehyde and dihydroxyacetone phosphate. This function is crucial for sustaining high glycolytic activity in ccRCC cells [80]. The downregulation of *ALDOB* by *STAT3* and *GATA2* **(Fig 4B)** promotes enhanced glycolytic flux, which is essential for meeting the increased energy demands of rapidly dividing tumor cells[152]. The growth and development of ccRCC are promoted by *ALDOB* downregulation both in vitro and in vivo [153]. Furthermore, its hypermethylation at CpG sites *cg13585586* and *cg14316227* is linked to an elevated risk of ccRCC. The HR value of 2.742 indicates that higher methylation levels of *ALDOB* might be involved in tumor progression (**Table 2**). This gene’s positive correlation with immune cells, like T cells, suggests its involvement in immune evasion and modulation, which can contribute to tumor development. This association implies the gene may support tumor growth by creating a microenvironment conducive to immune escape [152] (**S1 Fig in S1 File**). The gene *ENO2* plays a pivotal role in the *glycolytic pathway* (**Table 1**), converting 2-phosphoglycerate to phosphoenolpyruvate. This reaction is a crucial step in the production of ATP, which is essential for the energy demands of rapidly proliferating cancer cells. In ccRCC, the upregulation of *ENO2* is regulated by *FOXC1* **(Fig 4B)**, which promotes glycolysis and supports the Warburg effect—a metabolic shift observed in many cancers where cells rely on glycolysis for energy production even in the presence of oxygen [154]. This adaptation allows ccRCC cells to thrive in a hypoxic microenvironment by maintaining energy production and supporting tumor growth [155]. *ENO2* shows protective effects through hypomethylation, with CpG sites *cg02531908* and *cg22977254* having hazard ratios of 0.424 and 0.488, indicating a decreased risk of ccRCC (**Table 2**). It has negative correlations with immune cells like B cells and dendritic cells. This suggests it might be involved in reducing immune cell presence, possibly helping the tumor escape immune detection detection and promoting tumor progression [156]. *UMOD* influences renal illnesses including familial juvenile hyperuricemic nephropathy and medullary cystic kidney disease, which can lead to renal cancer [157]_._ *UMOD* is involved in potassium ion homeostasis and stress response (**Table 1**) [158]. In ccRCC, *UMOD*’s expression is regulated by *STAT3* and *FOXC1* **(Fig 4B)**, which enhances its role in maintaining ionic balance and managing cellular stress [159].*UMOD* helps ccRCC cells adapt to the challenging tumor microenvironment by regulating the release of extracellular vesicles, which can affect neighboring cells and modulate the immune response [159]. Its exhibits both protective and risk-associated methylation patterns in ccRCC. Hyper-methylation at CpG site *cg07817806* is associated with a higher HR of 2.062, suggesting a potential role in increased risk, while other sites show complex interactions with disease risk (**Table 2**). It positively correlates with CD8+ T cells and dendritic cells, suggesting a role in supporting cytotoxic T cell infiltration and antigen presentation, which may help ccRCC cells modulate the immune environment to survive [160] (**S1 Fig in S1 File**). By controlling the metabolism of aldehydes, the key gene *ALDH6A1* might have an impact on detoxification procedures, intermediate metabolism, and cellular redox balance, all of which might accelerate the tumor’s development in kidney. In ccRCC, *NFIC* and *GATA2* regulate *ALDH6A1* expression **(Fig 4B)**, enhancing its capacity to manage oxidative stress and detoxify harmful metabolites, which is crucial in the tumor environment [161]. This *gene* modulates the methylmalonyl-CoA metabolic pathway (**Table 1**), which impacts cancer stem cells (CSCs) in ccRCC [162]. It shows a significant risk association with ccRCC, with hypermethylation at CpG site cg14849468 yielding a hazard ratio of 3.099. This suggests that increased methylation of *ALDH6A1* could contribute to the development or worsening of ccRCC (**Table 2**). *ALDH6A1* could be a key player in immune modulation within the tumor microenvironment. The positive correlation with T cell CD8+ infiltration hints at a possible role in enhancing cytotoxic immune responses, which might impact tumor progression or response to immunotherapies in ccRCC [163] (**S1 Fig in S1 File**). According to a study, the key *gene SLC12A3* is implicated in the formation of ccRCC and is suggested as a biomarker for ccRCC diagnosis and treatment [164]. Additionally, the expression of *SLC12A1* is strongly correlated with patient survival duration in renal carcinoma [150]. Both *SLC12A3* and *SLC12A1* are essential for regulating ion transport in ccRCC (**Table 1**). *SLC12A3* is regulated by *STAT3* and *GATA2* **(Fig 4B)**, maintains potassium and chloride balance (**Table 1**), which is crucial for cellular homeostasis under stress and hypoxia. Similarly, *SLC12A1* is regulated by *NFIC*, *GATA2* and *FOXC1***(Fig 4B)**, ensures ionic balance and cellular volume, thereby supporting tumor cell survival and aggressive behavior [159]. *SLC12A1* shows a protective effect, as hypomethylation at CpG sites cg07915221 and cg14930674 is associated with hazard ratios of 0.442 and 0.422, which suggest a reduced risk of ccRCC. Similarly, *SLC12A3* shows hypomethylation associations with reduced risk of ccRCC. CpG sites cg01191774 and cg03455024 have hazard ratios of 0.295 and 0.432, suggesting a protective effect against the disease (**Table 2**). Furthermore, *SLC12A1* Shows strong positive correlation with CD8+ T cells and negative with macrophages and neutrophils, indicating its role in promoting cytotoxic T cell activity and potentially reducing macrophage-mediated immune suppression in ccRCC. *SLC12A3* Positively correlates with both CD8+ and CD4+ T cells and dendritic cells, pointing to its involvement in enhancing T cell-mediated immunity and antigen presentation, which could be crucial for anti-tumor responses in ccRCC [165] (**S1 Fig in S1 File**). The Plasminogen (*PLG*), a blood zymogen produced and involved in anti-tumor growth because it inhibits angiogenesis [166]. It is involved in the *complement and coagulation cascades* (**Table 1**), contributing to a pro-thrombotic environment that supports tumor growth and metastasis. *STAT3* regulates *PLG* expression **(Fig 4B)**, promoting a thrombotic state that facilitates tumor cell migration and invasion in ccRCC [167]. It is associated with an increased risk of ccRCC through hypermethylation of CpG sites. Specifically, methylation at *cg04181478* and *cg08531365* results in hazard ratios of 2.109 and 2.834, respectively, indicating that elevated *PLG* methylation correlates with a higher likelihood of disease progression (**Table 2**). *PLG* positively correlates with CD8+ T cells and dendritic cells, suggesting it supports cytotoxic T cell infiltration and antigen presentation, aiding ccRCC cells in modulating the immune environment for survival. Its negative association with macrophages indicates a complex role in selectively enhancing or suppressing immune pathways, which may influence the tumor microenvironment and affect disease progression [168] (**S1 Fig in S1 File**). *SERPINA5*, also known as antithrombin, modulates thrombin activity within the *coagulation cascade* (**Table 1**). According to reports *SERPINA5* plays a protective function against tumor formation, invasiveness, and metastasis and is dysregulated in renal, breast, prostate, liver, and ovarian malignancies [169]. Its expression is regulated by *NFIC* **(Fig 4B)**, which influences its levels and effects coagulation and immune responses. By contributing to a pro-thrombotic environment, *SERPINA5* supports ccRCC progression and metastasis, aiding in the tumor’s ability to evade immune surveillance and promote aggressive behavior. It also shows a potential protective effect against ccRCC, with hypomethylation at CpG sites *cg16937611* and *cg18731460* linked to decreased risk. The HR value of 0.462 suggests that lower methylation of *SERPINA5* could be associated with reduced disease susceptibility (**Table 2**). *SERPINA5* may facilitate immune evasion in ccRCC by promoting CD4+ T cell and B cell responses while suppressing CD8+ T cell, macrophage, and neutrophil activity. This imbalance could create a tumor microenvironment that supports tumor growth and survival [170] (**S1 Fig in S1 File**).

In order to explore KGs-guided possible pharmacological treatments for ccRCC, we estimated the binding affinity scores between 110 meta-drug molecules and KGs mediated receptors. We then identified the top 10 therapeutic agents (Irinotecan, Imatinib, Telaglenastat, Olaparib, RG-4733, Sorafenib, Sitravatinib, Cabozantinib, Abemaciclib, and Dovitinib) as the most viable candidate drugs for repurposing in ccRCC **(Fig 5A and S8 Table in S1 File)**. Subsequently, we compared the effectiveness of these ten chemical molecules against 30 different independent receptors that supported our results **(Fig 5B and S9 Table in S1 File)**. Many literature studies individually corroborated our hypothesis that this potential drug molecules may be efficacious against the treatment of ccRCC [55,171–187]. Among the ten proposed drugs, seven—namely Irinotecan (DB00762), Imatinib (DB00619), Sorafenib (DB00398), Cabozantinib (DB08875), Olaparib (DB09074), Abemaciclib (DB12187), and Dovitinib (DB08911)—are FDA-approved for various cancer treatments. According to Qi Zhang and colleagues, Olaparib which is a PARP inhibitor, may be a potentially useful treatment strategy for kidney cancers and it is FDA approved for ovarian, breast, and pancreatic cancers [172]. Imatinib, a potent PDGFR (platelet-derived growth factor receptor) inhibitor, has been utilized in the treatment of advanced renal cancer. By targeting and inhibiting the PDGFR, imatinib helps to impede tumor growth and progression, offering therapeutic benefits in managing this aggressive form of cancer [188]. The FDA recommends first-line treatments for ccRCC patients with anti-angiogenic-CPI regimens: axitinib plus avelumab or pembrolizumab, cabozantinib plus nivolumab, or lenvatinib plus pembrolizumab. [55,174]. According to the Drug Bank database, RG-4733 is an experimental medication being investigated for cancer (accesssion number DB11870). This new gamma secretase inhibitor, crucial for cleaving and activating Notch, is being investigated as an anti-cancer drug [175]. The investigation of sitravatinib is being conducted in the clinical research NCT03680521 (Neoadjuvant Sitravatinib in Combination With Nivolumab in Patients With Clear Cell Renal Cell Carcinoma) [176]. In patients with RCC, telaglenastat, a small-molecule glutaminase inhibitor, shows limited single-agent efficacy. However, in metastatic RCC (mRCC) patients, cabozantinib or everolimus—known to affect glucose metabolism—were tested in combination with telaglenastat in a phase Ib study. For extensively pretreated mRCC patients, the TelaE and TelaC combinations demonstrated excellent clinical activity and tolerability [177]. Irinotecan was initially approved for treating metastatic colorectal cancer [189]. Sorafenib [190], Cabozantinib [185] and Dovitinib [186] are both FDA-approved treatments for advanced ccRCC. These three drugs have been validated through extensive laboratory research and clinical studies. Abemaciclib is approved for treating some people with advanced or metastatic hormone receptor (HR)-positive, HER2-negative breast cancer that has progressed after hormone therapy [187]. We found that Imatinib, Sorafenib, Cabozantinib, and Dovitinib are FDA-approved treatments for advanced ccRCC. RG-4733 and Sitravatinib are currently in clinical trials for ccRCC. Irinotecan, Olaparib, Abemaciclib, and Telaglenastat are FDA-approved for other cancers.

Although the drug repurposing approach offers promising avenues for ccRCC treatment, several challenges and limitations must be acknowledged. The clinical relevance of binding affinity scores from in silico analyses does not always directly translate to therapeutic efficacy in patients due to factors like drug bioavailability, pharmacokinetics, and off-target effects. The tumor microenvironment in ccRCC, characterized by hypoxia and immune evasion, may also impact the effectiveness of repurposed drugs. To validate the drug molecules computationally, we conducted ADME/T analysis and evaluated their drug-likeness. All ten medications demonstrated drug-like properties, meeting at least four of Lipinski’s rule of five criteria **(S11 Table in S1 File),** and exhibited favorable ADME/T characteristics, such as high Human Intestinal Absorption (HIA) levels between 68.00% to 100%, and no carcinogenic effects and no carcinogenic effects **(Table 3).** Toxicity analyses revealed that these compounds are likely non-toxic, possess favorable drug-like characteristics, and are appropriate for oral consumption. However, clinical studies are necessary to validate the efficacy and safety of these repurposed drugs in ccRCC patients. Therefore, the findings of this study could serve as valuable resources for the diagnosis and treatment of ccRCC.

## 5. Conclusion

This *in-silico* study disclosed ccRCC-causing eight key genes (*PLG*, *ENO2*, *ALDOB*, *UMOD*, *SLC12A1*, *SLC12A3*, *SLC12A1*, and *SERPINA5*) through statistical LIMMA, survival probability and protein-protein interaction networks analyses. The pan-cancer analysis with KGs based on TCGA database also showed the significant association of KGs with different subtypes of kidney cancers including ccRCC. The gene-set enrichment study with the GO-term showed that KGs are significantly associated with some biological processes (potassium ion homeostasis, positive regulation of fibrinolysis), molecular function (heparin binding, sodium:potassium:chloride symporter activity), cellular components (apical plasma membrane, extracellular exosome) and KEGG pathways (Metabolic pathways, Complement and coagulation cascades) that are also associated with ccRCC. The gene regulatory network (GRN) analysis revealed four TFs proteins FOXC1, GATA2, NFIC, and STAT3 as the top-ranked transcriptional regulatory factors of KGs, and five miRNAs hsa-mir-124-3p, hsa-mir-34a-5p, hsa-mir-27a-3p, hsa-mir-335-5p, and hsa-mir-200b-3p as their post-transcriptional regulators. Finally, KGs-guided ten candidate drug molecules (Irinotecan, Imatinib, Telaglenastat, Olaparib, RG-4733, Sorafenib, Sitravatinib, Cabozantinib, Abemaciclib, and Dovitinib) were recommended by using molecular docking and ADME/T analysis, where seven drugs molecules (Irinotecan, Imatinib, Olaparib, Dactinomycin, Sorafenib, Nexavar and Cabozantinib) have been approved by FDA and the rest three molecules are under investigation for different cancers. However, the fundings of this study require experimental validation in wet-lab for taking a better treatment plan against ccRCC.

## Supporting information

S1 File Association of KGs with immune cells in ccRCC. **S1 Table.** The gene expression profile datasets that were analyzed in this study. **S2 Table.** Collection of ccRCC related candidate drug agents from published articles and other sources. **S3 Table.** Collection of proposed key genes from published articles. **S4 Table**. List of upregulated and downregulated common DEGs (cDEGs) of ccRCC in 9 datasets (GSE16441, GSE53757, GSE66270, GSE66272, GSE16449, GSE76351, GSE66271, GSE71963 and GSE36895). **S5 Table.** List of screened cDEGs (scDEGs) from cDEGs through survival analysis. **S6 Table.** List of key genes (KGs) from PPI network based on different topological measures. **S7 Table.** Pan-cancer analysis of KGs. **S8Table.** Docking/ (binding affinity) scores (kcal/mol) between the proposed target genes/proteins (receptors) and top ordered 50 candidate drugs (out of 327). **S9 Table.** Docking/ (binding affinity) scores (kcal/mol) between the proposed drugs and 30 independent receptors(published). **S10 Table.** The 3-dimension view of strong binding interactions between targets and drugs. **S11 Table.** Drug-likeness profile of top-ranked ten drugs.
(DOCX)
