# Supplementary material for: Screening of differential gene expression patterns through survival analysis for diagnosis, prognosis and therapies of clear cell renal cell carcinoma
Source: PLoS One. 2024 Sep 30;19(9):e0310843. doi: 10.1371/journal.pone.0310843 (PMC11441673; doi:10.1371/journal.pone.0310843)
Supplement: S1 File — S1 Table. The gene expression profile datasets that were analyzed in this study. S2 Table. Collection of ccRCC related candidate drug agents from published articles and other sources. S3 Table. Collection of proposed key genes from published articles. S4 Table. List of upregulated and downregulated common DEGs (cDEGs) of ccRCC in 9 datasets (GSE16441, GSE53757, GSE66270, GSE66272, GSE16449, GSE76351, GSE66271, GSE71963 and GSE36895). S5 Table. List of screened cDEGs (scDEGs) from cDEGs through survival analysis. S6 Table. List of key genes (KGs) from PPI network based on different topological measures. S7 Table. Pan-cancer analysis of KGs. S8Table. Docking/ (binding affinity) scores (kcal/mol) between the proposed target genes/proteins (receptors) and top ordered 50 candidate drugs (out of 327). S9 Table. Docking/ (binding affinity) scores (kcal/mol) between the proposed drugs and 30 independent receptors(published). S10 Table. The 3-dimension view of strong binding interactions between targets and drugs. S11 Table. Drug-likeness profile of top-ranked ten drugs. (DOCX) [file pone.0310843.s001.docx]

Screening of differential gene expression patterns through survival analysis for diagnosis, prognosis and therapies of clear cell renal cell carcinoma

Supplementary File

| **Table of Contents** | **Pages** |
| --- | --- |
| **S1 Table :** Description of gene-expression profile datasets that were analyzed in this study | **2** |
| **S2 Table.** Collection of ccRCC related candidate drug agents from published articles and others sources | **3** |
| **S3 Table.** Collection of proposed key genes from published articles. | **4-5** |
| **S4 Table.** List of upregulated and downregulated common DEGs (cDEGs) of ccRCC in 9 datasets (GSE16441, GSE53757, GSE66270, GSE66272, GSE16449, GSE76351, GSE66271, GSE71963 and GSE36895) | **5** |
| **S5 Table.** List of screened cDEGs (scDEGs) from cDEGs through survival analysis | **6-7** |
| **S6 Table.** List of key genes (KGs) from PPI network based on different topological measures | **7** |
| **S7 Table.** Pan-cancer analysis of KGs | **7-8** |
| **S8 Table.** Docking/(binding affinity) scores (kcal/mol) between the proposed target genes/proteins (receptors) and top ordered 50 candidate drugs (out of 327). | **9-10** |
| **S9 Table.** Docking/(binding affinity) scores (kcal/mol) between the proposed drugs and 30 independent receptors(published). | **10-11** |
| **S10 Table.** The 3-dimension view of strong binding interactions between targets and drugs | **11** |
| **S11 Table.** Drug-likeness profile of top-ranked ten drugs | **12** |
| **S1 Figure**. Association of sKGs with immune cells in ccRCC | **13-14** |
| **References** | **15-18** |

| S1 Table: The gene expression profile datasets that were analyzed in this study | | | | |
| --- | --- | --- | --- | --- |
| GEO Datasets | Country | Platform | Case | Control |
| GSE16441 | USA | GPL8659Agilent Human miRNA Microarray Rel12.0  Link:<https://www.ncbi.nlm.nih.gov/geo/query/acc.cgi?acc=GSE16441> | 17 | 17 |
| GSE53757 | USA | GPL570 [HG-U133_Plus_2] Affymetrix Human Genome U133 Plus 2.0 Array  Link:<https://www.ncbi.nlm.nih.gov/geo/query/acc.cgi?acc=GSE53757> | 17 | 17 |
| GSE66270 | Germany | GPL570[HG-U133_Plus_2] Affymetrix Human Genome U133 Plus 2.0 Array  Link: <https://www.ncbi.nlm.nih.gov/geo/query/acc.cgi?acc=GSE66270> | 14 | 14 |
| GSE66272 | Germany | GPL570[HG-U133_Plus_2] Affymetrix Human Genome U133 Plus 2.0 Array  Link: <https://www.ncbi.nlm.nih.gov/geo/query/acc.cgi?acc=GSE66272> | 27 | 27 |
| GSE16449 | USA | GPL6480 Agilent-014850 Whole Human Genome Microarray 4x44K G4112F (Probe Name version)  Link: <https://www.ncbi.nlm.nih.gov/geo/query/acc.cgi?acc=GSE16449> | 53 | 17 |
| GSE76351 | Russia | GPL11532[HuGene-1_1-st] Affymetrix Human Gene 1.1 ST Array [transcript (gene) version]  Link:<https://www.ncbi.nlm.nih.gov/geo/query/acc.cgi?acc=GSE76351> | 12 | 12 |
| GSE66271 | Germany | GPL570 [HG-U133_Plus_2] Affymetrix Human Genome U133 Plus 2.0 Array  Link: <https://www.ncbi.nlm.nih.gov/geo/query/acc.cgi?acc=GSE66271> | 13 | 13 |
| GSE71963 | Japan | GPL6480 Agilent-014850 Whole Human Genome Microarray 4x44K G4112F (Probe Name version)  Link: <https://www.ncbi.nlm.nih.gov/geo/query/acc.cgi?acc=GSE71963> | 32 | 16 |
| GSE36895 | USA | GPL570[HG-U133_Plus_2] Affymetrix Human Genome U133 Plus 2.0 Array  Link: <https://www.ncbi.nlm.nih.gov/geo/query/acc.cgi?acc=GSE36895> | 51 | 23 |

| S2 Table. Collection of ccRCC related candidate drug agents from published articles | |
| --- | --- |
| Articles | **Drug agents** |
| [1] | Clemizole, pentolonium, dioxybenzone, Prestwick-691, metoprolol |
| [2] | gallium nitrate, cladribine, amonafide |
| [3] | Sunitinib, Bevacizumab, pazopanib, Temsirolimus, Sorafenib, Everolimus |
| [4] | Monensin, quercetin, fenbufen |
| [5] | Everolimus, Sunitinib, Temsirolimus, Bevacizumab, pazopanib, Sorafenib |
| [6] | Josamycin, capecitabine, bevacizumab |
| [7] | Sunitinib, sorafenib, pazopanib, tivozanib, axitinib |
| [8] | FTI-277, KIN001-270, PD-173074 , Pazopanib, EHT-1864, Gefitinib, A832234, KOBE2602 , Albociclib. |
| [9] | Abemaciclib, Adavosertib, AGS-16C3F, Aldesleukin, Atezolizumab  Avelumab, Axitinib, Batiraxcept, Belzutifan, Benzalkonium, Bevacizumab, Cabozantinib, Cadonilimab, Camrelizumab, Carboplatin, Carotuximab, Casdozokitug, Cediranib, Ceralasertib, Cyclophosphamide, Cyclosporine, Dactinomycin, Daratumumab, Denosumab, Dostarlimab Dovitinib, Doxorubicin, Durvalumab, Entinostat, Enzalutamide, Erlotinib, Etoposide, Everolimus, Fludarabine, Fludeoxyglucose (18F), Girentuximab, Guadecitabine, Human interleukin-2, Imatinib, Indium, Interferon alfa-2b, Ioflupane I-123, Ipilimumab, Irinotecan, Ivuxolimab, Ixabepilone, Lutetium Lu-177, Mavorixafor, MEDI0680, Menthol, Mipsagargin, Mycophenolate mofetil, Niraparib, Nivolumab, Olaparib, Panobinostat, Patupilone, Pazopanib, Pembrolizumab, PT-2385, RG-4733, RO7502175, Savolitinib, Selenomethionine, Sintilimab, Sirolimus, Sitravatinib, Sorafenib, Spartalizumab, Sunitinib, Talazoparib, Tandutinib, Telaglenastat, Temsirolimus, Tremelimumab, Vincristine, Vorinostat |
| [10] | Afinitor, Afinitor Disperz, Aldesleukin, Alymsys, Avastin, Avelumab, Axitinib, Bavencio, Belzutifan, Bevacizumab, Cabometyx, Cabozantinib-S-Malate, Everolimus, Fotivda, IL-2, Inlyta, Interleukin-2, Ipilimumab, Keytruda, Lenvatinib Mesylate, Lenvima , Mvasi , Nexavar Nivolumab, Opdivo, Pazopanib Hydrochloride, Pembrolizumab, ProleukinSorafenib Tosylate, Sunitinib Malate, Sutent, Temsirolimus, Tivozanib Hydrochloride  Torisel, Votrient, Welireg, Yervoy , Zirabev . |
| [11] | Afinitor, aldesleukin, Alymsys, Avastin, Avelumab, Axitinib, Bavencio, Bevacizumab, Cabometyx, cabozantinib, Capecitabine, Erlotinib , Fotivda, Ipilimumab, Keytruda, Lenvatinib, Lenvima, Medroxyprogesterone, Mvasi, Nexavar, Nivolumab, Opdivo, Pazopanib, Peginterferon alfa-2b , Proleukin, Severolimus, Sorafenib, Sunitinib, temsirolimus, Tivozanib, Torisel, Vegzelma, Votrient, Yervoy, Zirabev. |

| **S3 Table.** List of key genes (KGs) collected published articles | | |
| --- | --- | --- |
| **Article reference** | **Key genes (KGs)** | **Published top-ranked key genes (KGs)** |
| [12] | APOB, IGFBP1, CP, ENAM, MFI2, CHRDL1, IL6, TF, FGA, FGG | Common genes in at least 3 articles:  ALB, ALDH6A1, ALDOB, BIRC5, CASR, CCNB2, CCND1  CEP55, CXCL12, CXCR4, EGF  EGFR, ENO2, FBP1, FOXM1  HMGCS2, HSD11B1,KIF20A  KNG1, MELK, MYC, OGDHL  PCK1, PECAM1, PLG, PTPRC  RRM2, SLC12A1, TOP2A  TPX2, TYROBP, UBE2C  VSIG4 |
| [13] | HRG, FABP1, ALDOB, PCK1, HAO2, CASR, PLG, and HMGCS2, SERPINE1 and TYROBP |  |
| [14] | APCDD1L, GJB6, CASP5, SLN, HSD11B1, PPARGC1A, ZPLD1, SLC22A12, SLC22A6, HMGCS2, CPA4, ADGRV1, GPAT3, PAEP, MZB1, RORB, IGLL5, OGDHL, AQP9, LDHD, FDCSP, HSD11B2, TNFSF13B, FREM1, FCRL5, POU2AF1, MUC20, VSIG4, RAP1GAP, MIXL1, GREM1, PAH, and SLC22A8 |  |
| [15] | C1QA, C1QB, C1QC, CCND1 and EGF |  |
| [16] | SLC34A1, SLC12A3, SLC12A1, PLG, and ENO2, LOX |  |
| [17] | TOP2A, MYC, ALB, CDK1, VEGFA, MMP9, PTPRC, CASR, EGFR and PTGS2 |  |
| [18] | UCN, PLG, FOXM1, HRH2 |  |
| [19] | PLG, FTCD, SLC34A1, HAO2, AGXT2, PCK1, HMGCS2, VWF, TIMP1, ICAM1,  CXCR4,  EGF, CAV1, CCND1, PECAM1, MME, ALB, CXCL12, CDH1, PROM1, ICAM1, PTPRC |  |
| [20] | PCOLCE, P4HB, COL6A2 and COL6A3 |  |
| [21] | C3, CXCR4, CCl4, ACKR3, KIF20A, CCNB2, CDCA8, CCL28, S1PR5, and CCL20 |  |
| [22] | CRYBB1, RIMBP3C, CEACAM4, HAMP, and LYL1 |  |
| [23] | ALDOB, MIOX, GPX3and MT1G |  |
| [24] | ALDOB, EFHD1, and ESRRG |  |
| [25] | ENO2, CCND1, PLT1, PLG and VWF |  |
| [26] | RPIA, G6PD, PSAT1, ENO2, HK3, IDH1, PDK4, PGM2, PGK1, FBP1, OGDH, SUCLA2, and SUCLG2 |  |
| [27] | RIPK4, TNF, CDC42, KNG1, PTPN11, KITLG, PTGS2, SYK, IGF1R, EPO, SERPINE1, FLT1, AURKB, GNA13, DLG2, ACTN2, CHEK1, FGF8, CD80 and MCHR2. |  |
| [28] | ALDH6A1, ALDH1L1, GLRX5, ALDH1A3, and GSTM3, SHMT1 |  |
| [29] | SUCLG1, PCK2, GLDC, SLC12A1, ATP1A1, PDHA1 |  |
| [30] | ABAT, ALDH6A1, CHDH, EPHX2, ETNK2, and FBP1. |  |
| [31] | CCNB2, CDC20, CEP55, KIF20A, TOP2A and UBE2 |  |
| [32] | COL4A5, ABCB1, NR3C2and PLG |  |
| [33] | PADI1, ATP6V0D2, DPP6, C9orf135 and PLG |  |
| [34] | CTLA4, DLGAP5, PLK1, CD44, BIRC5, MAD2L1, HLA-DRA, and HLA-G |  |
| [35] | ALB, VEGFA, TOP2A , EGFR,EGF ,EHHADH ,MYC ,CD44 ,GLDC ,ALDH7A1, ENO2, CSF1R ,ALDH3A2 ,ALDH1B1 ,ALDH6A1 |  |
| [36] | ITGAX, LAPTM5, and SERPINE1 |  |
| [37] | KNG1, ALDOB, C1QB, NPTX2, and UMOD |  |
| [38] | EGFR, FLT1, and EDN1 |  |
| [39] | ENO2, HK2, PFKP, SLC2A3, PDK1, SLC16A1, ALDOB, PKLR, PFKFB2, G6PC, PCK1, FBP1, PC, SUCLG1. |  |
| [40] | ABCB4, DPEP1, IL4I1, ENO2, PLD4, CEL, HSD11B2, ACADSB, ELOVL2, LPA, and PIK3R6 |  |
| [2] | TOP2A, BIRC5, BUB1, MELK, RRM2, and TPX2 |  |
| [41] | CCNA2, RRM2, FOXM1, CEP55, AURKB, NUF2, PTTG1, UBE2C, BIRC5, KIF20A, CCNB2, NCAPG, TPX2, KIF4A,HJURP, CDCA8 and CDCA3 |  |
| [42] | OXGR1, MAPK1, GNG2, LCK, ITGB2, HLA-DRB1, KIF20A, GNG10, GNB4, and HLA-DRA |  |
| [43] | PTPRC, TGFB1, EGF, MYC, ITGB2, CTSS, FN1, CCL5, KNG1, and CD86 |  |
| [44] | ENPP3, NNMT, CYP2J2, SCD, HK2, HSD11B2, HMGCS2, HPD, HS6ST2, and ALDOB |  |
| [45] | EGF, KDR, CXCL12, REN, PECAM1, CDH5, THY1, WT1, PLAU and DCN |  |
| [46] | APOE, CASR, CTLA4, CXCL8, EGF, F2, KNG1, MMP9, and IL6. |  |
| [47] | CASP5, HSD11B1, VSIG4, HMGCS2, HSD11B2, and OGDHL |  |
| [48] | HSD11B2 |  |
| [49] | KIF20A |  |
| [50] | CCNB2, CDC20, CEP55, KIF20A, TOP2A and UBE2C |  |
| [51] | CENPE, KIF20A, KIF4A, MELK, NCAPG, NDC80, NUF2, TOP2A, TPX2 and UBE2C, ACADM |  |
| [52] | TOP2A |  |
| [53] | PTTG1, RRM2, TOP2A, UHRF1, CEP55, BIRC5, UBE2C, FOXM1 and CDC20 |  |
| [54] | TYROBP, BIRC5, BUB1B, CENPF, and MELK |  |
| [55] | KI67, BIRC5, TP53, CXCR4, and CA9 |  |
| [56] | AGPAT9, AQP7, HMGCS2, KLF15, MLXIPL, PPARGC1A |  |
| [57] | CXCL12, BDKRB2, ADCY7, CASR, KNG1 and LPAR5 |  |
| [58] | SLC6A3, NPTX2, TNFAIP6, NDUFA4L2, ENPP3, FABP6, SPINK13, FXYD4, SLC12A1, KNG1, NPHS2, SLC13A3, GCGR, PLG |  |

| S4 Table. List of upregulated and downregulated cDEGs of ccRCC from 9 datasets (GSE16515, GSE15471, GSE71989) (GSE16441, GSE53757, GSE66270, GSE66272, GSE16449, GSE76351, GSE66271, GSE71963 and GSE36895) | |
| --- | --- |
| Downregulated cDEGs | **Upregulated cDEGs** |
| RALYL, SFRP1, TMEM61, KNG1, RASL11B, CA10  TFCP2L1, RALGPS1, FXYD4, PROM2, SUSD4, RAB25, ERBB4, SLC4A1, FAM3B, ACPP, ATP6V0A4, MAN1C1, DNASE1, PLPPR1, TFAP2B, CALB1, TMPRSS2, SLC4A9, CCDC181, ADGRF1 | ANGPTL4, VEGFA, ZNF395, TNFAIP6, NDUFA4L2, IGFBP3, SLC16A3, PLK2, EHD2, ENO2, LPCAT1, BHLHE41, HILPDADDB2, PPP1R13L, PYGL, AHNAK2, HK2, NNMT, DGCR5, ITGA5, C3, NRP2, LOX, CAV1, SCD. CLCNKB, AQP2, NR0B2, SH3GL2, EPB41L4B, ATP6V0D2HRG, ATP6V1B1, NELL1, GPAT3, SLC12A3, MPP7, NOS1PTH1R, PCP4, C16orf89, FECH, SOST, UMOD, APEH, NPHS1**,** CRHBP, PTGER3, IYD, MTURN, NR1I3, SLC13A3, MFSD4A**,** GGT6, FGF1, TMEM178A, ENPP6, ACSF2, WNK4, XPNPEP2**,** TSPAN33, RHBG, ALDH6A1, TNNT2, CYP17A1, CYP2B6**,** F11, GATA3, OCLN, ABAT, PRLR, SLC4A11, MT1G, TRPM3**,** DIO1, PLA2R1, WDR72, NPHS2, TGFBR3, MTCH2, TUBAL3**,** ACADSB, PLG, ATP6V0E2, ASS1, ALDH4A1, KLK6, ALDOB, SERPINA5,SLC12A1, HS6ST2, BMPR1B, FAM169A, ERP27, TMPRSS4, HYKK, HEPACAM2, KCNJ10, CLCNKA, FGF9, PACRG, GABRA2, ATP6V1C2, TMEM52B, KCNJ1, SUCLG1 |

-

| S5 Table. List of screened cDEGs (scDEGs) from cDEGs through survival analysis | |
| --- | --- |
| Screened cDEGs (scDEGs) | **Logrank p value** |
| ZNF395 | 0.00096 |
| TNFAIP6 | 4.4e−05 |
| SLC16A3 | 0.00029 |
| ENO2 | 0.019 |
| PPP1R13L | 0.011 |
| PROM2 | 0.00059 |
| NNMT |  |
| C3 | 4.1e−05 |
| NR1I3 | 0.00049 |
| SERPINA5 | 0.0036 |
| RASL11B | 0.0046 |
| RALGPS1 | 0.037 |
| SUSD4 | 5.7e−07 |
| RAB25 | 0.017 |
| SLC12A1 | 0.037 |
| FAM169A | 0.0011 |
| HEPACAM2 | 0.01 |
| ATP6V1C2 | 1.2e−09 |
| SUCLG1 | 2.6e−05 |
| EPB41L4B | 0.00044 |
| ATP6V0D2 | 0.0066 |
| SLC12A3 | 0.018 |
| MPP7 | 2.1e−11 |
| PTH1R | 3.6e−05 |
| FECH | 1.2e−05 |
| APEH | 0.0041 |
| CRHBP | 2.6e−08 |
| PTGER3 | 0.027 |
| IYD | 1.4e−06 |
| MTURN | 1.7e−09 |
| GGT6 | 0.061 |
| FGF1 | 0.00017 |
| TMEM178A | 0.024 |
| RHBG | 0.0019 |
| ALDH6A1 | 1.8e−10 |
| F11 | 0.0043 |
| OCLN | 4.5e−05 |
| ABAT | 0.00013 |
| PRLR | 0.013 |
| MT1G | 0.0018 |
| TRPM3 | 1.5e−09 |
| WDR72 | 2.2e−13 |
| TGFBR3 | 0.00029 |
| MTCH2 | 0.002 |
| PLG | 0.00011 |
| ALDOB | 3.7e−06 |
| HYKK | 0.002 |
| TMEM52B | 0.024 |
| SOST | 0.043 |
| UMOD | 0.046 |
| CYP2B6 | 0.0075 |
| DIO1 | 0.0029 |
| CLCNKA | 0.00016 |
| ACADSB | 4.3e−08 |

| S6 Table. List of key genes (KGs) from PPI network based on different topological measures | | | | | | | |
| --- | --- | --- | --- | --- | --- | --- | --- |
| SN. | **KGs** | **Degree** | **Closeness** | **EPC** | **MNC** | **MCC** | **Betweenness** |
| 1 | ALDOB | 17 | 31.66666667 | 16.619 | 14 | 39 | 858.8811 |
| 2 | PLG | 13 | 29.08333333 | 15.386 | 10 | 39 | 450.5367 |
| 3 | UMOD | 11 | 27 | 15.228 | 10 | 41 | 297.3652 |
| 4 | SLC12A3 | 10 | 26.66667 | 14.249 | 9 | 25 | 267.0017 |
| 5 | SLC12A1 | 9 | 26.08333333 | 14.085 | 7 | 22 | 259.7466 |
| 6 | ENO2 | 9 | 26.41666667 | 13.412 | 7 | 20 | 277.9165 |
| 7 | SERPINA5 | 7 | 23.2 | 12.975 | 7 | 22 | 250.6289 |
| 8 | ALDH6A1 | 7 | 23.83333333 | 11.377 | 6 | 27 | 248.4963 |

| **S7 Table.** Pan-cancer analysis of KGs | | | | | | | | |
| --- | --- | --- | --- | --- | --- | --- | --- | --- |
| **Cancer Name** | **ALDOB** | **ALDH6A1** | **ENO2** | **SERPINA5** | **PLG** | **SLC12A1** | **SLC12A3** | **UMOD** |
| Breast invasive carcinoma | 9.77E-22 | 5.90E-19 | 4.23E-14 | 0.213959 | 0.292681 | 0.017571 | 0.000294 | 0.213959 |
| Cholangio carcinoma | 4.29E-08 | 1.02E-07 | 8.10E-07 | 0.000248 | 1.36E-05 | 0.014061 | 0.121887 | 0.000248 |
| Colon adenocarcinoma | 0.049479 | 7.54E-15 | 0.150213 | 8.24E-11 | 0.547625 | 0.166278 | 0.043032 | 8.24E-11 |
| Esophageal carcinoma | 0.096155 | 6.45E-05 | 0.030028 | 0.000436 | 0.004576 | 0.609932 | 0.009082 | 0.000436 |
| Glioblastoma multiforme | 0.001431 | 0.000634 | 0.000238 | 0.000681 | 0.001198 | 0.001084 | 0.271725 | 0.000681 |
| Head and Neck squamous cell carcinoma | 6.71E-09 | 9.97E-11 | 1.89E-06 | 0.464978 | 0.009829 | 0.001283 | 0.000566 | 0.464978 |
| HNSC+HPV | 0.343471 | 2.84E-08 | 1.14E-21 | 3.80E-05 | 0.550626 | 0.501402 | 0.000987 | 3.80E-05 |
| Kidney Chromophobe | 5.75E-13 | 1.54E-06 | 0.000694 | 2.10E-06 | 3.32E-14 | 2.98E-13 | 1.10E-12 | 2.10E-06 |
| Kidney renal papillary cell carcinoma | 1.02E-20 | 9.95E-35 | 3.21E-36 | 3.68E-42 | 9.17E-19 | 4.82E-42 | 4.84E-38 | 3.68E-42 |
| Kidney renal clear cell carcinoma | 1.84E-18 | 1.16E-15 | 2.15E-15 | 3.59E-19 | 1.12E-18 | 3.29E-20 | 6.66E-20 | 3.59E-19 |
| Liver hepatocellular carcinoma | 3.72E-14 | 8.92E-17 | 0.702491 | 0.019122 | 1.75E-17 | 0.083372 | 0.069047 | 0.019122 |
| Lung adenocarcinoma | 0.871937 | 0.277624 | 6.90E-06 | 0.014794 | 8.92E-10 | 0.000411 | 0.001346 | 0.014794 |
| Lung squamous cell carcinoma | 3.84E-09 | 8.41E-14 | 1.08E-12 | 0.045622 | 9.04E-15 | 0.410276 | 0.206955 | 0.045622 |
| Pheochromocytoma and Paraganglioma | 0.003288 | 0.028671 | 0.003173 | 0.003288 | 0.004927 | 0.618427 | 0.001772 | 0.003288 |
| Prostate adenocarcinoma | 0.354046 | 0.000272 | 2.85E-14 | 2.75E-19 | 0.003666 | 0.004862 | 0.792708 | 2.75E-19 |
| Rectum adenocarcinoma | 0.000302 | 9.91E-06 | 0.345869 | 0.000145 | 0.027902 | 0.005704 | 0.004854 | 0.000145 |
| Skin Cutaneous Melanoma | 0.646206 | 0.000175 | 0.171532 | 0.000937 | 0.80115 | 0.000471 | 0.000875 | 0.000937 |
| Thyroid carcinoma | 2.39E-05 | 4.02E-15 | 2.58E-06 | 1.13E-26 | 0.772785 | 0.396118 | 5.58E-05 | 1.13E-26 |
| Uterine Corpus Endometrial Carcinoma | 0.000267 | 0.048862 | 0.055671 | 0.573471 | 0.673978 | 0.029143 | 1.39E-06 | 0.573471 |

| **S8 Table:** Docking/(binding affinity) scores (kcal/mol) between the proposed target genes/proteins (receptors) and top ordered 40 candidate drugs Out of 107). | | | | | | | | | | | | |
| --- | --- | --- | --- | --- | --- | --- | --- | --- | --- | --- | --- | --- |
| **Drug** | **ALDH6A1** | **ALDOB** | **ENO2** | **SLC12A1** | **SLC12A3** | **NFIC** | **SERPINA5** | **GATA2** | **STAT3** | **UMOD** | **PLG** |  |
| **Irinotecan** | -10.6 | -9.7 | -9.5 | -8.6 | -8.3 | -8.4 | -8.1 | -8.5 | -8.5 | -7.9 | -7.4 |  |
| **Imatinib** | -9.9 | -9.6 | -9.1 | -8.8 | -8.4 | -8.1 | -8.9 | -8.3 | -8.7 | -7.6 | -7.1 |  |
| **Telaglenastat** | -9.4 | -8.5 | -9.2 | -9.5 | -8.9 | -8.8 | -8.5 | -7.4 | -8.6 | -8 | -7.7 |  |
| **Olaparib** | -9.8 | -8.8 | -9.3 | -8.4 | -8.5 | -8.3 | -9 | -7.8 | -8.4 | -7.9 | -6.9 |  |
| **Dactinomycin** | -9.7 | -9.5 | -9.4 | -9.5 | -8.3 | -8.7 | -8.2 | -7.7 | -7.6 | -9.1 | -6 |  |
| **RG_4733** | -9.5 | -9.4 | -8.7 | -9.3 | -9.1 | -9.2 | -8.7 | -7.3 | -8.1 | -7.1 | -7.1 |  |
| **Sorafenib** | -9.1 | -8.5 | -8.4 | -8.5 | -9.1 | -9.9 | -8.9 | -7.9 | -7.4 | -7.4 | -6.8 |  |
| **Nexavar** | -9.1 | -8.5 | -8.3 | -8.1 | -8.2 | -9.9 | -8.8 | -7.9 | -7.2 | -8.1 | -6.8 |  |
| **Sitravatinib** | -9.4 | -8.5 | -7.8 | -8.4 | -8.2 | -8.8 | -9 | -7.5 | -7.9 | -6.9 | -7.3 |  |
| **Cabozantinib** | -9.4 | -8.4 | -8.2 | -8.3 | -8.6 | -8.7 | -8.2 | -8 | -8.7 | -6.8 | -7.6 |  |
| **Abemaciclib** | -9.6 | -8 | -9.2 | -8.2 | -8 | -8.7 | -8.7 | -7.5 | -7.8 | -7.2 | -6.7 |  |
| **Dovitinib** | -8.7 | -8.5 | -8.1 | -8.2 | -8.2 | -8.5 | -8 | -7.9 | -7.5 | -7 | -6.8 |  |
| **Cabozantinib_S_malate** | -9.6 | -8.4 | -8.2 | -8.8 | -9 | -8.6 | -8.2 | -7.9 | -6.7 | -6.8 | -7.6 |  |
| **Votrient** | -9.4 | -8.9 | -8.3 | -8.5 | -9.1 | -8.5 | -8.4 | -7.8 | -7 | -6.8 | -6.8 |  |
| **Josamycin** | -8.8 | -9.4 | -7.9 | -9.1 | -8.5 | -9.4 | -8.9 | -7.2 | -6.4 | -7.3 | -7 |  |
| **Niraparib** | -9.3 | -8.8 | -8.4 | -8.7 | -8.6 | -8.9 | -8.2 | -7.8 | -7.9 | -7.4 | -6.8 |  |
| **Pazopanib_Hydrochloride** | -9.4 | -8.5 | -8.3 | -8.2 | -8 | -8.5 | -8.4 | -7.8 | -7.3 | -6.8 | -6.7 |  |
| **Talazoparib** | -9.1 | -8.4 | -7.4 | -9.1 | -8 | -8.2 | -8.6 | -8.2 | -7.3 | -7.3 | -6.5 |  |
| **Inlyta** | -9.2 | -8.7 | -7.8 | -8.7 | -8.5 | -8.1 | -8.7 | -7.5 | -8.2 | -7.4 | -6.8 |  |
| **Guadecitabine** | -8.7 | -9.1 | -7.8 | -9 | -8.7 | -8.2 | -8.1 | -6.3 | -7.2 | -6.8 | -6.6 |  |
| **Fotivda** | -8.5 | -8.7 | -8 | -8.4 | -8.9 | -8.5 | -7.9 | -7.4 | -7.2 | -7.2 | -6.8 |  |
| **tivozanib** | -8.8 | -8.5 | -8.1 | -8.3 | -8.1 | -8.3 | -7.9 | -7.5 | -7 | -7.2 | -6.7 |  |
| **Etoposide** | -9.5 | -8.6 | -8.1 | -8.6 | -8.5 | -9.1 | -7.8 | -7.8 | -6.2 | -6.2 | -6.7 |  |
| **Axitinib** | -8.6 | -8.7 | -7.6 | -8.6 | -8.4 | -8.1 | -8.5 | -7.5 | -8.2 | -7.4 | -6.8 |  |
| **Tivozanib_Hydrochloride** | -8.5 | -8.7 | -8 | -8.9 | -8.5 | -8.4 | -8 | -7.2 | -7.2 | -7.2 | -6.8 |  |
| **Lenvatinib_Mesylate** | -8.9 | -8.7 | -8.4 | -8.6 | -8.3 | -8.4 | -8.5 | -7.1 | -7.9 | -6.7 | -6.9 |  |
| **Doxorubicin** | -8.5 | -8.3 | -8 | -8.2 | -8.1 | -8.3 | -7.5 | -7 | -7.1 | -6.7 | -6.7 |  |
| **Adavosertib** | -8.7 | -8.4 | -8 | -8.4 | -8.1 | -8.7 | -8.3 | -7.5 | -6.7 | -6.9 | -6.2 |  |
| **Temsirolimus** | -8.4 | -9.1 | -8.4 | -8.6 | -8.4 | -8.7 | -8.3 | -6.5 | -6.2 | -7 | -6.6 |  |
| **Lenvatinib** | -8.8 | -8.5 | -8.2 | -8.3 | -8.3 | -8.6 | -7.9 | -7.4 | -7.9 | -6.7 | -6.4 |  |
| **Enzalutamide** | -8.2 | -8.8 | -8.2 | -8.5 | -8.3 | -8.5 | -8 | -6.6 | -6.8 | -6.3 | -6.4 |  |
| **Entinostat** | -8.6 | -8.5 | -7.9 | -8.1 | -8.4 | -8.4 | -8 | -7.4 | -8 | -6.8 | -6.5 |  |
| **Defibrotide_sodium** | -9.4 | -7.8 | -7.8 | -7.6 | -7.9 | -7.9 | -7.5 | -7.2 | -6.5 | -7.6 | -6.3 |  |
| **Savolitinib** | -8.7 | -8.2 | -7.6 | -8.6 | -8.4 | -8.8 | -7.5 | -6.9 | -7.3 | -6.6 | -6 |  |
| **Cediranib** | -9.4 | -7.7 | -7.6 | -7.6 | -7.3 | -8.1 | -7.6 | -6.8 | -7.3 | -6 | -6.1 |  |
| **Bendroflumethiazide** | -9.2 | -7.5 | -7.2 | -7.4 | -7.4 | -7.7 | -7.4 | -7.5 | -6.2 | -6.4 | -6.8 |  |
| **Fenretinide** | -7.7 | -7.9 | -7.7 | -8.9 | -8.2 | -7.2 | -8.3 | -7.1 | -6.5 | -7 | -6.5 |  |
| **Torisel** | -7.3 | -8.9 | -8.4 | -8.7 | -8.1 | -7 | -8.2 | -7.2 | -6.9 | -6.1 | -5.7 |  |
| **Everolimus** | -8.4 | -8.5 | -8.5 | -8.3 | -8.7 | -8.7 | -7.9 | -6 | -6 | -5.9 | -5.9 |  |
| **Sirolimus** | -9.5 | -7.7 | -8.3 | -7.4 | -7.8 | -8.1 | -7.6 | -7.9 | -6.2 | -6 | -5.1 |  |

| **S9 Table**. Docking/(binding affinity) scores (kcal/mol) between the proposed drugs and 30 independent receptors(published). | | | | | | | | | | |
| --- | --- | --- | --- | --- | --- | --- | --- | --- | --- | --- |
| **Drug** | Imatinib | Irinotecan | Olaparib | RG-4733 | Nexavar | Dactinomycin | Cabozantinib | Sitravatinib | Telaglenastat | Sorafenib |
| **HSD11B2** | -10.8 | -10.4 | -11 | -10.8 | -10.6 | -9.6 | -11 | -11.4 | -10.9 | -10.6 |
| **ALB** | -10.7 | -10.9 | -9.9 | -9.9 | -10.4 | -12.3 | -10.7 | -9.7 | -9.9 | -10.4 |
| **HMGCS2** | -9.4 | -9.9 | -9.3 | -9.9 | -10.1 | -10.4 | -9.9 | -9.6 | -10 | -10.3 |
| **PCK1** | -10.8 | -10 | -10.1 | -10.4 | -9.4 | -9.7 | -9.3 | -9 | -8.9 | -10.5 |
| **SLC12A1** | -10.2 | -9.7 | -9.7 | -9.8 | -9.3 | -10.4 | -9.1 | -9.7 | -8.9 | -9.5 |
| **ALDH6A1** | -9.9 | -9.8 | -10.4 | -9.5 | -9.1 | -9.7 | -8.7 | -8.8 | -10.4 | -9.7 |
| **TPX2** | -9.9 | -9.6 | -9.6 | -8.8 | -9.8 | -11 | -9.1 | -8.9 | -8.8 | -9.6 |
| **CASR** | -9.1 | -9.9 | -9 | -8.8 | -9.2 | -11 | -9 | -9 | -9.6 | -9 |
| **MELK** | -9.8 | -9.4 | -9.7 | -8.9 | -9 | -9.7 | -9.2 | -9.2 | -9.2 | -8.4 |
| **CCNB2** | -9.8 | -10.4 | -9.1 | -9.3 | -8.7 | -8.9 | -8.9 | -8.9 | -9 | -8.8 |
| **TOP2A** | -9 | -9.1 | -9.1 | -9.3 | -9.4 | -9.4 | -9.2 | -8.9 | -8.2 | -8.9 |
| **BIRC5** | -9 | -8.6 | -8.5 | -9.7 | -9.1 | -7.8 | -8.9 | -8.6 | -9.4 | -9.1 |
| **OGDHL** | -9 | -8.4 | -7.8 | -8.7 | -8.6 | -9.6 | -8.1 | -8.7 | -8.5 | -8.5 |
| **ALDOB** | -8.3 | -8.7 | -8 | -9.4 | -8.6 | -9.4 | -8.5 | -8.1 | -8.2 | -8.5 |
| **RRM2** | -9 | -8.5 | -8.8 | -9.5 | -8.7 | -8.4 | -8.2 | -7.7 | -7.1 | -9.2 |
| **FBP1** | -8.5 | -8.6 | -9.1 | -7.9 | -8.5 | -8.9 | -8.6 | -7.9 | -8.2 | -8.5 |
| **ENO2** | -8.2 | -8.6 | -9.4 | -8 | -8.4 | -9.7 | -8.1 | -8.6 | -7.3 | -8.3 |
| **CCND1** | -8 | -7.8 | -7.9 | -8 | -7.5 | -8.7 | -8 | -8.1 | -8.2 | -7.5 |
| **UBE2C** | -7.9 | -8.6 | -7.8 | -7.9 | -7.7 | -8 | -7.3 | -7.9 | -6.9 | -8.2 |
| **CXCL12** | -7.6 | -8 | -7.5 | -7.2 | -7.4 | -7.2 | -7.8 | -7.4 | -7.5 | -7.1 |
| **VSIG4** | -7.8 | -8.1 | -7.8 | -7.6 | -7.1 | -6 | -7.3 | -7.6 | -6.3 | -7.1 |
| **PLG** | -7.8 | -7.9 | -7.2 | -7.1 | -6.8 | -6.1 | -7.6 | -7.6 | -7.8 | -6.7 |
| **PTPRC** | -7.5 | -8.2 | -6.8 | -7.2 | -7.2 | -7.2 | -6.8 | -7.5 | -6.3 | -7.2 |
| **CEP55** | -7.3 | -7.6 | -7.7 | -7 | -7 | -6.9 | -6.9 | -7.2 | -7.2 | -6.9 |
| **FOXM1** | -7.6 | -7.5 | -7.6 | -7.4 | -7.4 | -7.4 | -7.4 | -7.4 | -6.4 | -6.6 |
| **EGFR** | -7.5 | -7 | -7.2 | -7.6 | -7.5 | -9.3 | -6.6 | -5.9 | -6.7 | -6.5 |
| **CXCR4** | -7.3 | -7.5 | -7.5 | -7 | -7.7 | -7.2 | -7.4 | -7.3 | -6.2 | -6.6 |
| **KIF20A** | -7.2 | -7.1 | -7 | -6.7 | -7.4 | -7.4 | -7.3 | -6.8 | -7.1 | -6.4 |
| **EGF** | -7.8 | -7.9 | -7.6 | -6.8 | -7.4 | -7.7 | -7.2 | -7.1 | -6.8 | -6.2 |
| **KNG1** | -7.5 | -7.6 | -7.8 | -6.1 | -7.6 | -7.8 | -7.9 | -7.1 | -7.5 | -6.1 |

| **S10 Table.** The 3-dimension view of strong binding interactions between targets and drugs | | | | |
| --- | --- | --- | --- | --- |
| **Protein and Ligand complex** | **Binding Affinity (kCal/ mol)** | **The 3d View**  **Complex** | **Target-Ligand Interaction** | **Interacting Amino Acids** |
| ALDH6A1  &  Irinotecan | -10.6 | 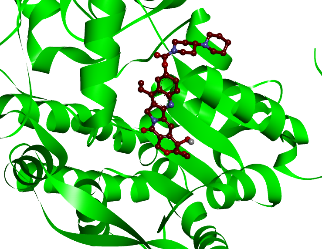 | 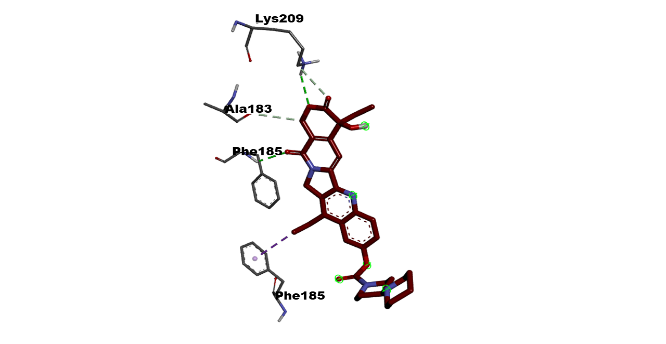 | LYS209, ALA183, PHE185, PHE419 |
| ALDOB  &  Imatinib | -9.6 | 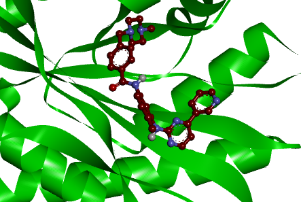 | 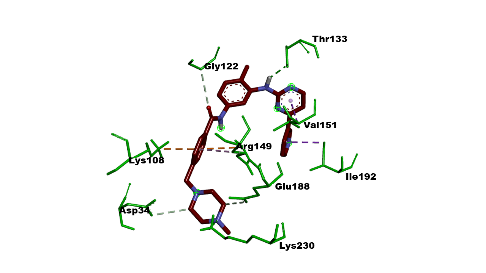 | THR123, GLY112, VAL151,ARG149, LYS108, GLU188, ILE192, GLU188, ASP34, LYS230 |
| ENO2  &  Telaglenastat | -9.4 | 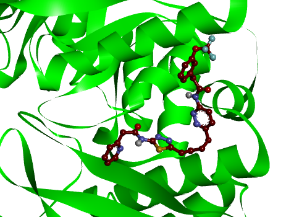 | 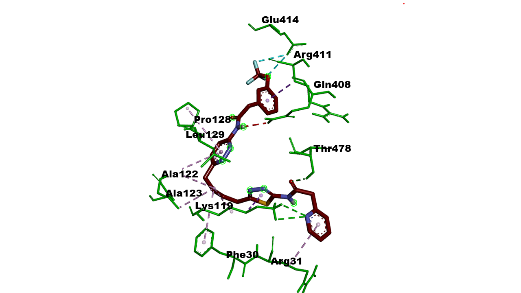 | GLU414, ARG411, GLN408, PRO128, LEU129, THR378, ALA122,ALA123, LYS129, PHE130, ARG31 |

| S11 Table. Drug-likeness profile of top-ranked ten drugs | | | | | | | | |
| --- | --- | --- | --- | --- | --- | --- | --- | --- |
| Compounds | Molecular weight | LogP | Water Solubility (ESOL) | GI absorption | Log Kp  (Cm/S) | Lipinski rule | | Synthetic accessibility |
|  |  |  |  |  |  | Follow | Violation |  |
| Imatinib | 493.615 | 4.5903 | -5.07 | High | -6.81 | 5 | 0 | 3.78 |
| Irinotecan | 586.689 | 4.0911 | -5.71 | High | -7.22 | 4 | 1 | 5.59 |
| Telaglenastat | 571.57 | 4.5495 | -5.12 | Low | -7.20 | 4 | 1 | 4.09 |
| Olaparib | 434.46 | 2.3474 | -3.70 | High | -7.60 | 5 | 0 | 3.15 |
| RG-4733 | 469.40 | 3.803 | -5.36 | High | -5.93 | 5 | 0 | 3.88 |
| Sorafenib | 464.831 | 5.5497 | -5.11 | Low | -6.25 | 5 | 0 | 2.87 |
| Sitravatinib | 629.689 | 6.5222 | -6.52 | Low | -6.47 | 3 | 2 | 4.38 |
| Cabozantinib | 501.514 | 5.5408 | -6.13 | High | -5.53 | 4 | 1 | 3.09 |
| Abemaciclib | 506.605 | 4.9369 | -5.36 | High | -6.66 | 4 | 1 | 3.87 |
| Dovitinib | 392.438 | 2.5445 | -3.66 | High | -7.53 | 5 | 0 | 3.20 |

| **S1 Figure**. Association of sKGs with immune cells in ccRCC |
| --- |
| 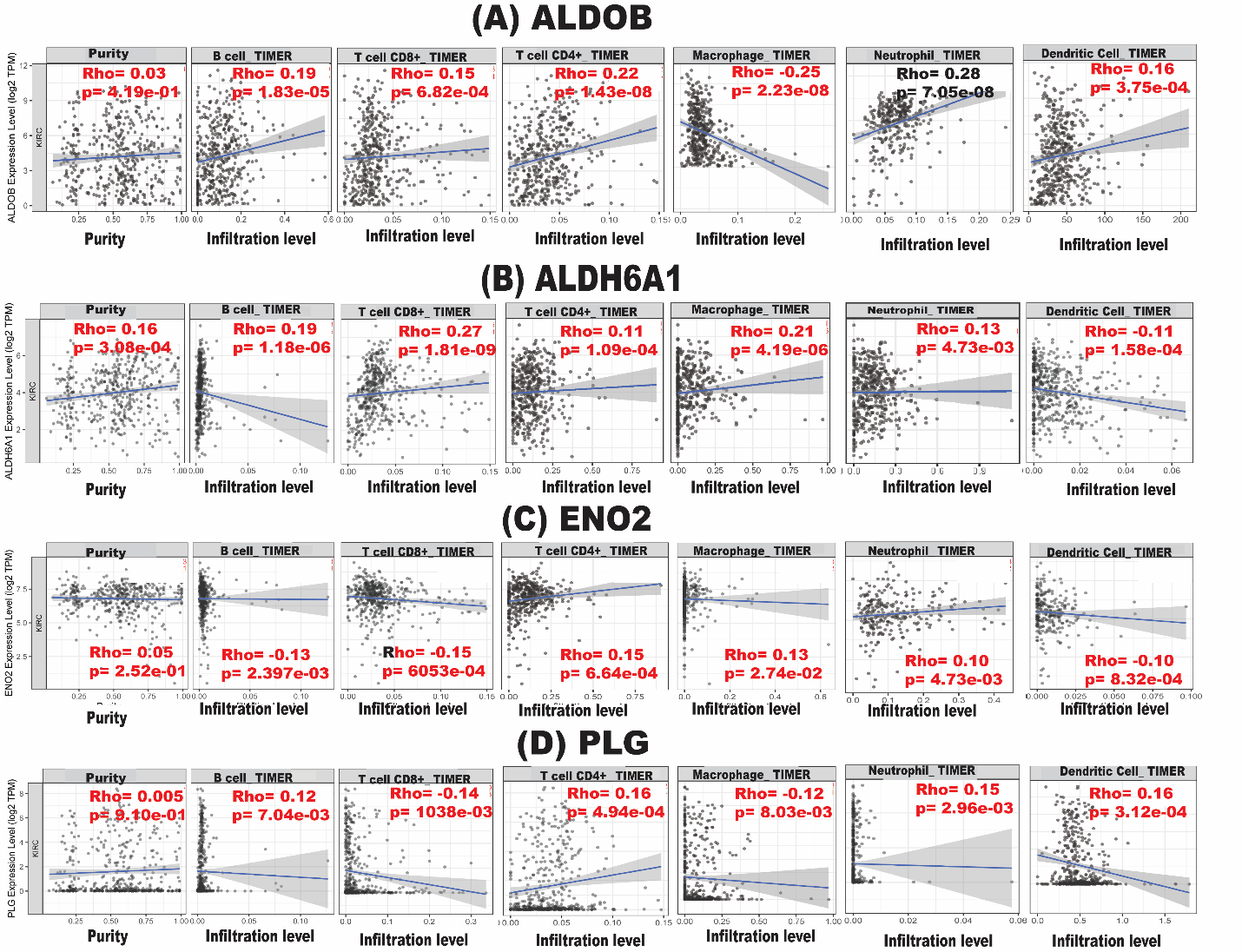 |

\

| **S1 Figure**. Association of sKGs with immune cells in ccRCC (C**ontinued**) |
| --- |
| 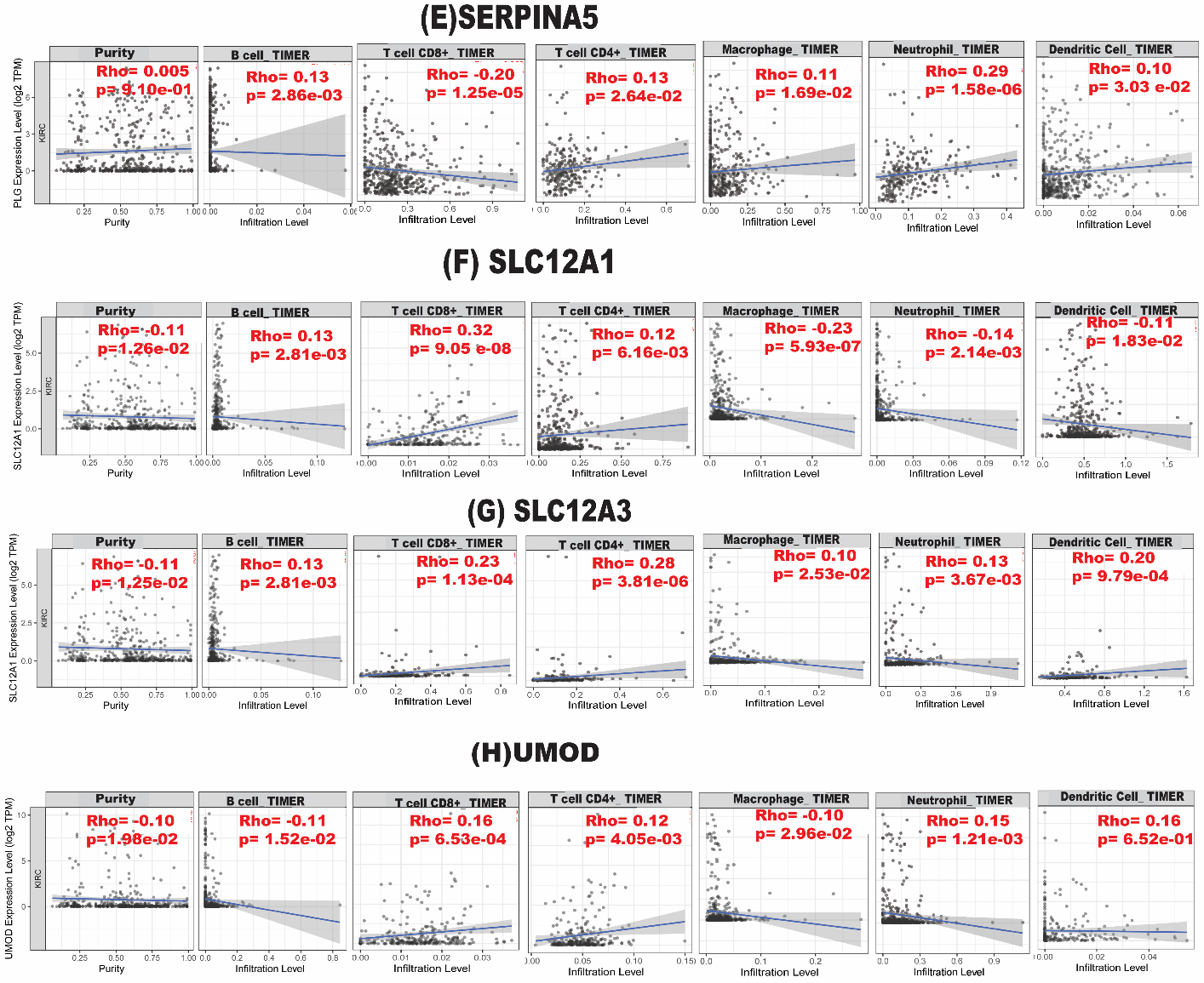 |

**Reference:**

[1] W. Li, X. Meng, H. Yuan, W. Xiao, and X. Zhang, “A Novel Immune-Related ceRNA Network and Relative Potential Therapeutic Drug Prediction in ccRCC,” *Front. Genet.*, vol. 12, no. January, pp. 1–13, 2022, doi: 10.3389/fgene.2021.755706.

[2] M. Lu, L. Xiao, B. Xu, and Q. Gao, “Identification of Novel Genes and Associated Drugs in AdvanceClear Cell Renal Cell Carcinoma by Bioinformatic Methods,” *Tohoku J. Exp. Med.*, vol. 258, no. 2, pp. 79–90, 2022, doi: 10.1620/tjem.2022.J059.

[3] M. Sun, G. Lughezzani, P. Perrotte, and P. I. Karakiewicz, “Treatment of metastatic renal cell carcinoma,” *Nat. Rev. Urol.*, vol. 7, no. 6, pp. 327–338, 2010, doi: 10.1038/nrurol.2010.57.

[4] G. F. Xiao, X. Yan, Z. Chen, R. J. Zhang, T. Z. Liu, and W. L. Hu, “Identification of a Novel Immune-Related Prognostic Biomarker and Small-Molecule Drugs in Clear Cell Renal Cell Carcinoma (ccRCC) by a Merged Microarray-Acquired Dataset and TCGA Database,” *Front. Genet.*, vol. 11, no. August, pp. 1–14, 2020, doi: 10.3389/fgene.2020.00810.

[5] M. W. Kramer, A. S. Merseburger, I. Peters, S. Waalkes, and M. A. Kuczyk, “Systemische und operative therapie des metastasierten nierenzellkarzinoms,” *Urol. - Ausgabe A*, vol. 51, no. 2, pp. 217–225, 2012, doi: 10.1007/s00120-011-2713-5.

[6] S. Bai *et al.*, “Construct a circRNA/miRNA/mRNA regulatory network to explore potential pathogenesis and therapy options of clear cell renal cell carcinoma,” *Sci. Rep.*, vol. 10, no. 1, pp. 1–15, 2020, doi: 10.1038/s41598-020-70484-2.

[7] E. Jonasch, M. B. Atkins, S. Chowdhury, and P. Mainwaring, “Combination of Anti-Angiogenics and Checkpoint Inhibitors for Renal Cell Carcinoma: Is the Whole Greater Than the Sum of Its Parts?,” *Cancers (Basel).*, vol. 14, no. 3, 2022, doi: 10.3390/cancers14030644.

[8] A. Jiang *et al.*, “A novel thinking: DDR axis refines the classification of ccRCC with distinctive prognosis, multi omics landscape and management strategy,” *Front. Public Heal.*, vol. 10, 2022, doi: 10.3389/fpubh.2022.1029509.

[9] DRUGBANK online, “Advanced Kidney Cancer-DRUGBANK online”, [Online]. Available: https://go.drugbank.com/indications/DBCOND0092155

[10] NIH, “NIH The Cancer Genome Atlas Program - NCI,” *Cancer Genome Atlas Progr.*, [Online]. Available: https://www.cancer.gov/ccg/research/genome-sequencing/tcga

[11] Drugs.com, “Drugs used to treat Renal Cell Carcinoma-Drugs.com,” *Drugs used to treat Ren. Cell Carcinoma*, [Online]. Available: https://www.drugs.com/condition/renal-cell-carcinoma.html

[12] G. A. Puzanov, “Identification of key genes of the ccRCC subtype with poor prognosis,” *Sci. Rep.*, pp. 1–10, 2022, doi: 10.1038/s41598-022-18620-y.

[13] P. Wu, T. Xiang, J. Wang, R. Lv, and G. Wu, “TYROBP is a potential prognostic biomarker of clear cell renal cell carcinoma,” vol. 10, pp. 2588–2604, 2020, doi: 10.1002/2211-5463.12993.

[14] E. Zhao, “Identification of Key Genes of Prognostic Value in Clear Cell Renal Cell Carcinoma Microenvironment and a Risk Score Prognostic Model,” vol. 2020, 2020.

[15] Z. Tian, C. Yuan, K. Yang, and X. Gao, “Systematic identification of key genes and pathways in clear cell renal cell carcinoma on bioinformatics analysis,” vol. 7, no. 5, 2019, doi: 10.21037/atm.2019.01.18.

[16] L. Peng *et al.*, “Screening of possible biomarkers and therapeutic targets in kidney renal clear cell carcinoma: Evidence from bioinformatic analysis,” *Front. Oncol.*, vol. 12, no. October, pp. 1–10, 2022, doi: 10.3389/fonc.2022.963483.

[17] L. Yuan, G. Zeng, L. Chen, G. Wang, X. Wang, and X. Cao, “Identification of key genes and pathways in human clear cell renal cell carcinoma ( ccRCC ) by co-expression analysis,” vol. 14, 2018, doi: 10.7150/ijbs.23574.

[18] S. Ma, Y. Ge, Z. Xiong, Y. Wang, L. Li, and Z. Chao, “A novel gene signature related to oxidative stress predicts the prognosis in clear cell renal cell carcinoma,” pp. 1–22, 2023, doi: 10.7717/peerj.14784.

[19] W. Zhai, H. Lu, S. Dong, J. Fang, and Z. Yu, “Identification of potential key genes and key pathways related to clear cell renal cell carcinoma through bioinformatics analysis,” *Acta Biochim. Biophys. Sin. (Shanghai).*, vol. 52, no. 8, pp. 853–863, 2020, doi: 10.1093/abbs/gmaa068.

[20] T. Zhong, X. Wang, and H. Wang, “Key Genes Associated With Prognosis and Metastasis of Clear Cell Renal Cell Carcinoma,” pp. 1–17, 2021.

[21] R. Peng, “Identification of Core Genes Involved in the Metastasis of Clear Cell Renal Cell Carcinoma,” pp. 13437–13449, 2020.

[22] Y. Peng, S. Dong, and H. Wang, “Key sunitinib- ­ related biomarkers for renal cell carcinoma,” no. May, pp. 6917–6930, 2021, doi: 10.1002/cam4.4206.

[23] J. Hu *et al.*, “Single-Cell Transcriptome Analysis Reveals Intratumoral Heterogeneity in ccRCC , which Results in Different Clinical Outcomes,” *Mol. Ther.*, vol. 28, no. 7, pp. 1658–1672, 2020, doi: 10.1016/j.ymthe.2020.04.023.

[24] H. Huang *et al.*, “Identification of Hub Genes Associated With Clear Cell Renal Cell Carcinoma by Integrated Bioinformatics Analysis,” *Front. Oncol.*, vol. 11, no. September, pp. 1–12, 2021, doi: 10.3389/fonc.2021.726655.

[25] T. Luo *et al.*, “Bioinformatic identification of key genes and analysis of prognostic values in clear cell renal cell carcinoma,” *Oncol. Lett.*, vol. 16, no. 2, pp. 1747–1757, 2018, doi: 10.3892/ol.2018.8842.

[26] Y. Zhang, M. Chen, M. Liu, Y. Xu, and G. Wu, “Glycolysis-Related Genes Serve as Potential Prognostic Biomarkers in Clear Cell Renal Cell Carcinoma,” vol. 2021, 2021.

[27] L. Bao *et al.*, “The Identification of Key Gene Expression Signature and Biological Pathways in Metastatic Renal Cell Carcinoma,” vol. 11, 2020, doi: 10.7150/jca.38379.

[28] X. Wei *et al.*, “Redox Metabolism-Associated Molecular Classification of Clear Cell Renal Cell Carcinoma,” vol. 2022, 2022.

[29] D. Gu, “Identifying the novel key genes in renal cell carcinoma by bioinformatics analysis and cell experiments CURRENT STATUS : UNDER REVIEW”, doi: 10.21203/rs.2.23818/v1.

[30] Q. Zhang, L. Ding, T. Zhou, Q. Zhai, and C. Ni, “A metabolic reprogramming- related prognostic risk model for clear cell renal cell carcinoma : From construction to preliminary application,” no. September, pp. 1–15, 2022, doi: 10.3389/fonc.2022.982426.

[31] L. Yuan *et al.*, “Co-expression network analysis identified six hub genes in association with progression and prognosis in human clear cell renal cell carcinoma (ccRCC),” *Genomics Data*, vol. 14, no. November, pp. 132–140, 2017, doi: 10.1016/j.gdata.2017.10.006.

[32] Y. Liu *et al.*, “Development of a four-gene prognostic model for clear cell renal cell carcinoma based on transcriptome analysis,” *Genomics*, vol. 113, no. 4, pp. 1816–1827, 2021, doi: 10.1016/j.ygeno.2021.04.005.

[33] Z. Zhang *et al.*, “Construction of a novel gene ‑ based model for prognosis prediction of clear cell renal cell carcinoma,” *Cancer Cell Int.*, pp. 1–18, 2020, doi: 10.1186/s12935-020-1113-6.

[34] X. Yu, H. Wu, H. Wang, H. Dong, and B. Gao, “Identification of 8 Feature Genes Related to Clear Cell Renal Cell Carcinoma Progression Based on Co-Expression Analysis,” pp. 113–124, 2022, doi: 10.1159/000520832.

[35] R. Li, L. E. I. Wang, X. Wang, R. X. I. N. Geng, N. Li, and X. I. U. H. Liu, “Identification of hub genes associated with outcome of clear cell renal cell carcinoma,” pp. 2846–2860, 2020, doi: 10.3892/ol.2020.11389.

[36] Y. Sui, K. Lu, and L. Fu, “Prediction and analysis of novel key genes ITGAX, LAPTM5, SERPINE1 in clear cell renal cell carcinoma through bioinformatics analysis,” *PeerJ*, vol. 9, pp. 1–21, 2021, doi: 10.7717/peerj.11272.

[37] Y. Yuan, J. Wang, L. Huang, and Y. Guo, “Bioinformatics identification of prognostic genes and potential interaction analysis in renal cell carcinoma,” 2023, doi: 10.21037/tcr-22-2242.

[38] S. Wang, Z. H. Yu, and K. Q. Chai, “Identification of CFTR as a novel key gene in chromophobe renal cell carcinoma through bioinformatics analysis,” *Oncol. Lett.*, vol. 18, no. 2, pp. 1767–1774, 2019, doi: 10.3892/ol.2019.10476.

[39] E. Sanders and S. Diehl, “Analysis and interpretation of transcriptomic data obtained from extended Warburg effect genes in patients with clear cell renal cell carcinoma,” *Oncoscience*, vol. 2, no. 2, pp. 151–186, 2015, doi: 10.18632/oncoscience.128.

[40] K. Li *et al.*, “A novel lipid metabolism gene signature for clear cell renal cell carcinoma using integrated bioinformatics analysis,” *Front. Cell Dev. Biol.*, vol. 11, no. February, pp. 1–14, 2023, doi: 10.3389/fcell.2023.1078759.

[41] J. yi Chen *et al.*, “Co-expression Network Analysis Identifies Fourteen Hub Genes Associated with Prognosis in Clear Cell Renal Cell Carcinoma,” *Curr. Med. Sci.*, vol. 40, no. 4, pp. 773–785, 2020, doi: 10.1007/s11596-020-2245-6.

[42] S. Akçay, “INTEGRATED NETWORK ANALYSIS OF THE POTENTIAL MOLECULAR BIOMARKERS AND KEY PATHWAYS IN CLEAR RENAL CELL CARCINOMA ( ccRCC ),” vol. 15, no. 3, pp. 342–351, 2021.

[43] E. Song *et al.*, “Identification of potential crucial genes associated with carcinogenesis of clear cell renal cell carcinoma,” *J. Cell. Biochem.*, vol. 119, no. 7, pp. 5163–5174, 2018, doi: 10.1002/jcb.26543.

[44] J. Xie, L. Cui, S. Pan, D. Liu, F. Liu, and Z. Liu, “Metabolic Understanding of the Genetic Dysregulation in the Tumor Microenvironment of Kidney Renal Clear Cell Carcinoma,” *Dis. Markers*, vol. 2022, 2022, doi: 10.1155/2022/6085072.

[45] Y. Xu, D. Kong, Z. Li, L. Qian, J. Li, and C. Zou, “Screening and identification of key biomarkers of papillary renal cell carcinoma by bioinformatic analysis,” *PLoS One*, vol. 16, no. 8 August, pp. 1–11, 2021, doi: 10.1371/journal.pone.0254868.

[46] J. Li *et al.*, “A Bioinformatic Analysis of Immune-Related Prognostic Genes in Clear Cell Renal Cell Carcinoma Based on TCGA and GEO Databases,” *Int. J. Gen. Med.*, vol. 15, pp. 325–342, 2022, doi: 10.2147/IJGM.S341801.

[47] D. Hu, M. Zhou, and X. Zhu, “Deciphering Immune-Associated Genes to Predict Survival in Clear Cell Renal Cell Cancer,” *Biomed Res. Int.*, vol. 2019, 2019, doi: 10.1155/2019/2506843.

[48] J. Wang, L. Tao, Y. Liu, H. Liu, X. Shen, and L. Tao, “Identification and validation of DLX4 as a prognostic and diagnostic biomarker for clear cell renal cell carcinoma,” *Oncol. Lett.*, vol. 25, no. 4, pp. 1–19, 2023, doi: 10.3892/ol.2023.13732.

[49] W. Xiao, K. Chen, H. G. Liang, and X. P. Zhang, “Identification of KIF20A as a tumor biomarker and forwarder of clear cell renal cell carcinoma,” *Chin. Med. J. (Engl).*, vol. 134, no. 17, pp. 2137–2139, 2021, doi: 10.1097/CM9.0000000000001331.

[50] L. Yuan *et al.*, “Co-expression network analysis identified six hub genes in association with progression and prognosis in human clear cell renal cell carcinoma (ccRCC),” *Genomics Data*, vol. 14, no. October, pp. 132–140, 2017, doi: 10.1016/j.gdata.2017.10.006.

[51] H. Zhang *et al.*, “Bioinformatic analysis identifies potentially key differentially expressed genes in oncogenesis and progression of clear cell renal cell carcinoma,” *PeerJ*, vol. 2019, no. 11, pp. 1–26, 2019, doi: 10.7717/peerj.8096.

[52] Y. Xiong *et al.*, “Identifying a novel biomarker TOP2A of clear Cell Renal Cell Carcinoma (ccRCC) associated with smoking by co-expression network analysis,” *J. Cancer*, vol. 9, no. 21, pp. 3912–3922, 2018, doi: 10.7150/jca.25900.

[53] Y. Luo, D. Shen, L. Chen, G. Wang, X. Liu, and K. Qian, “FOXM1_ageing_iii,” vol. 11, no. 16, 2019.

[54] B. Wan, Y. Yang, and Z. Zhang, “Identification of Differentially Methylated Genes Associated with Clear Cell Renal Cell Carcinoma and Their Prognostic Values,” *J. Environ. Public Health*, vol. 2023, p. 8405945, 2023, doi: 10.1155/2023/8405945.

[55] F. Petitprez, M. Ayadi, A. de Reyniès, W. H. Fridman, C. Sautès-Fridman, and S. Job, “Review of Prognostic Expression Markers for Clear Cell Renal Cell Carcinoma,” *Front. Oncol.*, vol. 11, no. April, 2021, doi: 10.3389/fonc.2021.643065.

[56] Y. Wang, J. Yang, Q. Zhang, J. Xia, and Z. Wang, “Extent and characteristics of immune infiltration in clear cell renal cell carcinoma and the prognostic value,” *Transl. Androl. Urol.*, vol. 8, no. 6, pp. 609–618, 2019, doi: 10.21037/tau.2019.10.19.

[57] H. Cui *et al.*, “Integrated bioinformatics analysis for the identification of potential key genes affecting the pathogenesis of clear cell renal cell carcinoma,” *Oncol. Lett.*, vol. 20, no. 2, pp. 1573–1584, 2020, doi: 10.3892/ol.2020.11703.

[58] S. Schrödter *et al.*, “Identification of the dopamine transporter SLC6A3 as a biomarker for patients with renal cell carcinoma,” *Mol. Cancer*, vol. 15, no. 1, pp. 1–10, 2016, doi: 10.1186/s12943-016-0495-5.
